# Time series analysis of low-concentration air pollution and hospital respiratory disease outpatient visits

**DOI:** 10.3389/fpubh.2025.1585086

**Published:** 2025-05-20

**Authors:** Yongxin Wang, Jingwen Chen, Quan Zhou, Shuling Kang, Yu Jiang, Jianjun Xiang, Jing Wu, Jin Li, Zhiwei Chen, Chuancheng Wu

**Affiliations:** ^1^Department of Preventive Medicine, School of Public Health, Fujian Medical University, Fuzhou, China; ^2^Fuzhou Center for Disease Control and Prevention, Fuzhou, China

**Keywords:** air pollution, respiratory diseases, time series analysis, low-concentration air pollution, disease risk

## Abstract

**Objective:**

The aim of this study was to analyze the effects of short-term exposure to low concentrations of air pollutants on the volume of respiratory outpatient visits in hospitals and their lagged effects.

**Methods:**

The study collected outpatient data from seven hospitals in Fuzhou City, air pollution data provided by the Fuzhou Environmental Monitoring Center Station, and meteorological data from the Fuzhou Meteorological Bureau for analysis from 2019 to 2022. Time series analysis was used to explore the relationship between air pollutants and meteorological factors and daily outpatient visits for respiratory diseases by constructing a generalized linear model (GLM).

**Results:**

From 2019 to 2022, the total outpatient volume of respiratory diseases in 7 hospitals in Fuzhou was 1,530,000, with pediatrics accounting for 72.44% and internal medicine accounting for 27.56%. Air pollutants such as PM_2.5_, PM_10_, NO_2_, and SO_2_ all had significant impacts on the total respiratory and pediatric respiratory outpatient volumes. NO_2_ and PM_10_ had the greatest impact on respiratory diseases on the day of pollution exposure or 1 day later, while SO_2_ and PM_2.5_ exhibited longer lag effects, with the most significant impact occurring at a lag period of 4–6 days. The impact of air pollution on pediatric respiratory disease outpatient visits was generally more significant than that on adult.

**Conclusion:**

Low concentrations of air pollution significantly impacted respiratory outpatient visits in Fuzhou, especially in children. Despite relatively good air quality, air pollution in low-pollution areas poses a public health risk, highlighting the need for targeted pollution control policies.

## 1 Introduction

Respiratory infections are one of the key public health issues with high incidence and mortality rates worldwide. According to the 2021 Global Burden of Disease Study, upper respiratory infections have become the most prevalent disease globally, with a total of 12.8 billion cases. Due to the impact of the COVID-19 pandemic, COVID-19 became the second leading cause of age-standardized death, resulting in 7.89 million deaths. Lower respiratory infections rank as the seventh leading cause of death worldwide (1). Therefore, the impact of respiratory infections on the human respiratory system is a global health concern.

Many respiratory infections are airborne, and air pollution is one of the most important risk factors for respiratory diseases (2). Air pollutants not only damage the human immune system through their chemical components, thereby compromising health, but they can also carry pathogens that enter the human body through the respiratory tract (3–5). Studies have found a positive correlation between the increase in confirmed cases of SARS-CoV-2 and the air quality index (6–8).

Existing research indicates that airborne particulate matter (PM), particularly smaller particles like PM₂.₅, poses significant health risks. These particles can penetrate both the upper and lower respiratory systems, reach the alveoli, and even enter other systems through the bloodstream. In addition to causing respiratory diseases, PM₂.₅ is closely associated with cardiovascular issues, strokes, and neurological disorders (9, 10).

Moreover, PM₂.₅ typically does not exist in isolation, but interacts with other pollutants such as PM_10_, NO₂, SO₂, and O₃, which can exacerbate airway inflammation. These inflammatory responses further damage respiratory tissues, significantly increasing the severity of respiratory diseases and the risk of conditions like chronic obstructive pulmonary disease (COPD) and asthma (11).

Furthermore, climate and air pollution are mutually influential and interdependent. Meteorological changes affect the activity of pathogens in the air and on surfaces, as well as the diffusion and deposition of pollutants, thereby influencing transmission (5, 12–14). For example, high wind speeds can accelerate the spread of pollutants, reducing their concentration and thereby lowering their harmful effects on the respiratory system (15). On the other hand, high humidity promotes the formation and settlement of aerosol particles, further reducing air pollution levels. In addition, stable weather conditions (such as high atmospheric pressure) may lead to the accumulation of pollutants, increasing the risk of cardiovascular and respiratory diseases (16). This effect is amplified when combined with air pollution, enhancing the body’s susceptibility (17). However, existing studies primarily focus on areas with high pollution exposure or the analysis of pollution sources. There has been less attention given to the direct link between low-concentration air pollution exposure and specific diseases, such as respiratory diseases (18, 19). The concentration-health effect is often higher at low concentrations than at high concentrations, as the health risks of low-level exposure are not always adequately addressed (20).

Therefore, this study aims to utilize hospital outpatient data, air pollution data, and meteorological data from Fuzhou between 2019 and 2022 to conduct a time-series analysis of the association between low-concentration air pollution exposure and respiratory outpatient visits. By capturing both the immediate and delayed effects of pollutant concentration fluctuations on health, this study will offer a refined evaluation of health impacts. It will provide new perspectives on understanding the effects of low-concentration air pollution on public health and serve as an important reference and epidemiological basis for the adjustment of public health policies in Fuzhou and similar areas with low pollution concentrations.

The forest coverage rate in Fuzhou, Fujian Province, China, reaches 58.41%, and the air quality is excellent. Therefore, this study explored the effects of air pollutants exposure on total hospital outpatient visits and respiratory outpatient visits in Fuzhou City, a low pollution exposure area, by analyzing hospital outpatient data, air pollutant and meteorological data simultaneously to provide an epidemiological basis for governmental measures in low pollution areas.

## 2 Materials and methods

### 2.1 Source of information

#### 2.1.1 Air pollution data

The air pollution data (PM_2.5_, PM_10_, NO_2_, SO_2_, O_3_, CO) from January 2019 to December 2022 were sourced from six national control points (Fujian Normal University, Gushan, Ziyang, Kuaian, Wusi North Road, Yangqiao West Road) and one municipal control point (Fuzhou No. 29 Middle School) monitored by the Fuzhou Environmental Monitoring Center Station. These monitoring stations provide real-time data on local pollutant concentrations. Among the monitoring sites, Wusi North Road, Yangqiao West Road, Ziyang, Fujian Normal University, and Fuzhou No. 29 Middle School are located within the city’s third ring road, while the Kuaian monitoring site is located outside the third ring road in Mawei Town, and the Gushan site is situated within the Gushan Scenic Area. These environmental monitoring points cover most areas of Fuzhou City. To balance the representativeness of the data and the ability to detect localized effects, the study area was divided into several sub-regions for stratified analysis, and the daily average concentrations of each pollutant were calculated separately.

#### 2.1.2 Meteorological data

The meteorological data during the same period were sourced from the Fuzhou Meteorological Bureau, including daily average temperature (°C), daily average atmospheric pressure (hPa), daily average relative humidity (%), and daily average wind speed (m/s).

#### 2.1.3 Outpatient data

All data were systematically organized and cleaned using Microsoft Excel 2016. SPSS 26.0 software was employed to perform a descriptive analysis of air pollutant data, meteorological data, and respiratory disease outpatient data within the urban area of Fuzhou from January 2019 to December 2022. The data concerning air pollutants, meteorological indicators, and daily outpatient visits for respiratory diseases were statistically summarized using measures such as mean standard error, minimum, maximum, and quartiles. The outpatient data related to respiratory diseases were categorized into internal medicine respiratory disease outpatient visits and pediatric respiratory disease outpatient visits to assess whether there were significant differences between the two groups. Respiratory diseases were further classified into acute upper respiratory tract infections, other acute lower respiratory tract infections, influenza and pneumonia, various diseases of the upper respiratory tract, and chronic lower respiratory diseases. By analyzing the daily outpatient data for respiratory diseases collected from various hospitals, the outpatient composition ratio for different types of respiratory diseases was determined.

### 2.2 Statistical method

#### 2.2.1 Descriptive analysis

Microsoft Excel 2016 was used to organize and clean all data. Descriptive analysis of the air pollution data, meteorological data, and respiratory disease outpatient data from Fuzhou’s urban area (January 2019 to December 2022) was conducted using SPSS 26.0 software. The air pollution, meteorological indicators, and daily outpatient data for respiratory diseases were statistically described using means, standard errors, minimum values, maximum values, and quartiles. Respiratory disease outpatient data were divided into two categories: outpatient visits for respiratory diseases in internal medicine and outpatient visits for pediatric respiratory diseases, and significant differences between these two groups were analyzed. Respiratory diseases were categorized into five subtypes: acute upper respiratory tract infections, other acute lower respiratory tract infections, influenza and pneumonia, other upper respiratory tract diseases, and chronic lower respiratory tract diseases. Based on the daily outpatient data for respiratory diseases from each hospital, the composition ratio of outpatient visits for each subtype was calculated.

#### 2.2.2 Correlation analysis and factor analysis

Spearman correlation analysis was performed on the air pollutant data, meteorological data and outpatient data through R (version 4.4.3) to assess the correlation between air pollutants, meteorological factor variables and respiratory outpatient visits and to make graphs, if the correlation is too high it means that there is a strong covariance and it is not suitable to be included in the model. Stepwise regression analysis was performed on the above data using SPSS 26.0 software to find out the factors that have an impact on the volume of respiratory outpatient visits.

#### 2.2.3 Modeling time series

In order to explore the model with better results in terms of the effects of meteorological factors and air pollution on outpatient respiratory diseases, we compare the autoregressive integrative sliding average model (ARIMA) and the generalized linear model (GLM), which are widely used in the field of time series analysis, with the general form of the ARIMA model as ARIMA (p, d, q), p represents the number of autoregressive terms, d is the difference order, and q is the sliding average. Its optimal parameter combination is found to be ARIMA (1, 2) in this study. Ljung-Box Q-test for white noise of residuals is used. It is calculated based on the autocorrelation coefficients of multiple lags and measures the overall autocorrelation of the residuals. Values were used to test whether the statistic was significant. If *p*<0.05, the null hypothesis can be rejected (the residuals are white noise). In Supplementary Table S1, the *p*-value for lag 1 is 0.0082, which is less than 0.05, indicating that there are structures or patterns in the residuals that are not captured by the model, and that there may be some degree of autocorrelation, which does not satisfy the white noise test, and that the model does not fit the data adequately. In order to quantify the uncertainty of the predictive model, the data were also evaluated using Mean Error (ME), Root Mean Squared Error (RSME), Mean Absolute Error (MAE), and Mean Absolute Percentage Error (MAPE) as shown in Supplementary Table S1, and it was found that that the degree of its error is large. Due to the poor fitting effect and prediction of ARIMA model, we finally used Generalized Linear Models (GLM) (21) based on Poisson distribution with the ability to control data over-discretization and autocorrelation in the study of the health effects of air pollution, based on the date, atmospheric pollutant concentration, meteorological conditions, day of the week, and the number of days of the week. Health effect indicators (daily respiratory outpatient visits) were used to create time series data to analyze the effect of air pollutants on respiratory disease outpatient visits. Correlation analysis revealed a strong correlation between daily mean barometric pressure and daily mean temperature (*r* = −0.81), and daily mean barometric pressure was eventually excluded from the model to avoid covariance. Adjustment variables for inclusion in the final model were determined based on the principle of minimizing the value of the quasi-Akaike information criterion (Q-AIC) (22). For temperature (T) and relative humidity (RH), a degree of freedom of 4 was used (23, 24). The degrees of freedom (df) for dates were 7 df per year (25). The GLM formula was as follows:


$$\text{logE}\;(\mathrm{Yt})=\beta \mathrm{Zt}+\mathrm{ns}(\text{time},7^{*}4)+\mathrm{ns}(\mathrm{Xt},4)+\mathrm{DOW}+ɑ.$$

In this model, E(Yt) represents the expected value of respiratory outpatient visits on the t-th day (in persons). β denotes the exposure-response relationship coefficient. Zt is the observed concentration of atmospheric particulate pollution on the t-th day. ns() refers to the natural spline function used to fit the long-term and seasonal trends of the time series, with df representing its degrees of freedom. Time is the date variable. Xt represents the observed meteorological factors on the t-th day, including average temperature and average relative humidity. DOW is a dummy variable reflecting the day-of-week effect. *α* is the constant intercept term.

This study aims to investigate the short-term health effects of pollutants by observing the days within a 7-day lag period, analyzing the sensitivity of various pollutants to both the same-day outpatient visits and the visits with a lag of 1 to 7 days. Specifically, the excess risk (ER) of outpatient visits for each 10 μg/m^3^ increase in pollutant concentration was calculated. The concentrations of pollutants at lag0 to lag7 (0–7 days) were sequentially incorporated into the model to calculate the ER and 95% confidence intervals (CI) for each 10 μg/m^3^ increase in pollutant concentration, analyzing the lag effects individually. The GLM model plot was generated using ggplot2 in R software. In this study, a *p*-value of ≤0.05 was considered statistically significant.

#### 2.2.4 Sensitivity analysis

To evaluate the stability of the findings of this study, a sensitivity analysis was further conducted on the model by modifying the df values of the covariates, either increasing or decreasing the original df values by 1. Finally, the impact on the results was assessed based on the extent of change in their corresponding effect values.

## 3 Results

### 3.1 Descriptive statistics of air pollutants and meteorological factors

The general descriptive statistics for air pollutants and meteorological variables in Fuzhou from 2019 to 2022 are presented in Table 1. The mean concentrations of PM₁₀, PM₂.₅, NO₂, SO₂, CO, and O₃ were 37.01 ± 16.61 μg/m^3^, 19.32 ± 9.804 μg/m^3^, 18.23 ± 8.285 μg/m^3^, 3.96 ± 0.97 μg/m^3^, 588.38 ± 145.06 μg/m^3^, and 88.96 ± 30.09 μg/m^3^, respectively. As shown in Supplementary Table S2, the annual average concentrations of the six pollutants exhibited a declining trend over the study period, except for an abrupt increase in the annual average concentration of O₃-8 h in 2022.

**Table 1:** Basic characteristics of respiratory outpatient visits, air pollutants and meteorological factors in Fuzhou from 2019 to 2022.

Variables	Mean ± SD	Minimum	Median	Maximum	IQR
Respiratory outpatient visits					
Total	1047.23 ± 500.05	112.00	965.00	3351.00	658.50
Pediatrics	758.58 ± 407.04	83.00	686.00	2537.00	579.00
Internal Medicine	288.65 ± 147.89	17.00	272.00	1103.00	184.00
Air pollutants					
PM_10_ (μg/m^3^)	37.01 ± 16.61	5.25	35.38	145.57	21.24
PM_2.5_ (μg/m^3^)	19.32 ± 9.80	2.50	17.75	75.86	11.70
NO_2_ (μg/m^3^)	18.23 ± 8.29	3.57	16.86	49.75	10.44
SO_2_ (μg/m^3^)	3.96 ± 0.97	2.00	3.88	7.71	1.29
CO (mg/m^3^)	588.38 ± 145.06	212.50	585.71	1100.00	187.50
O_3_-8h (μg/m^3^)	88.96 ± 30.09	20.71	87.43	186.57	42.46
Meteorological factors					
Atmospheric pressure (hPa)	1006.57 ± 7.96	985.00	1007.00	1028.00	13.00
Temperature (°C)	21.55 ± 6.89	5.10	21.50	34.00	12.30
Relative Humidity (%)	73.30 ± 12.94	37.00	72.00	100.00	18.00
Wind Speed (m/s)	2.11 ± 0.72	0.60	2.00	7.10	0.90

The daily average concentrations of PM₂.₅ and PM₁₀ showed a general “W-shaped” trend across seasons, as illustrated in Supplementary Figure S1, with lower levels observed in summer and autumn, and higher levels in winter and spring.The annual average values of key meteorological factors in Fuzhou were as follows: atmospheric pressure 1006.57 ± 7.96 hPa, temperature 21.55 ± 6.89°C, relative humidity 73.30 ± 12.94%, and wind speed 2.11 ± 0.72 m/s. As shown in Supplementary Figure S2, temperature and atmospheric pressure exhibited clear seasonal patterns, although atmospheric pressure showed multiple sharp declines between March and October of 2022. Relative humidity and wind speed fluctuated markedly throughout the study period.

The compliance rates of air pollutants with respect to concentration standards were calculated based on the six pollutants (Supplementary Table S3). As shown in Table 2, the concentrations of NO₂, SO₂, and CO meet the national Class I standards, while the daily average concentrations of PM₁₀, PM₂.₅, and O₃ generally comply with the national Class II standards, indicating that the air quality in Fuzhou is relatively good. However, according to the WHO standards, the compliance rates for PM₁₀, PM₂.₅, and O₃ decrease to 81.86, 77.75, and 64.48%, respectively.

**Table 2:** The compliance rate of air pollutants in Fuzhou from 2019 to 2022.

Air pollutants	Total days (d)	China (attainment rate)		WHO attainment rate (%)
		First-level (%)	Second-level (%)	
PM_10_ (μg/m^3^)	1,461	81.86	100.00	81.86
PM_2.5_ (μg/m^3^)	1,461	92.54	99.93	77.75
NO_2_ (μg/m^3^)	1,461	100.00	100.00	–
SO_2_ (μg/m^3^)	1,461	100.00	100.00	100.00
CO (mg/m^3^)	1,461	100.00	100.00	–
O_3_-8h (μg/m^3^)	1,461	64.48	99.04	64.48

### 3.2 Basic situation of outpatient visits for respiratory diseases and the composition ratio of daily outpatient visits

**Figure 1:**
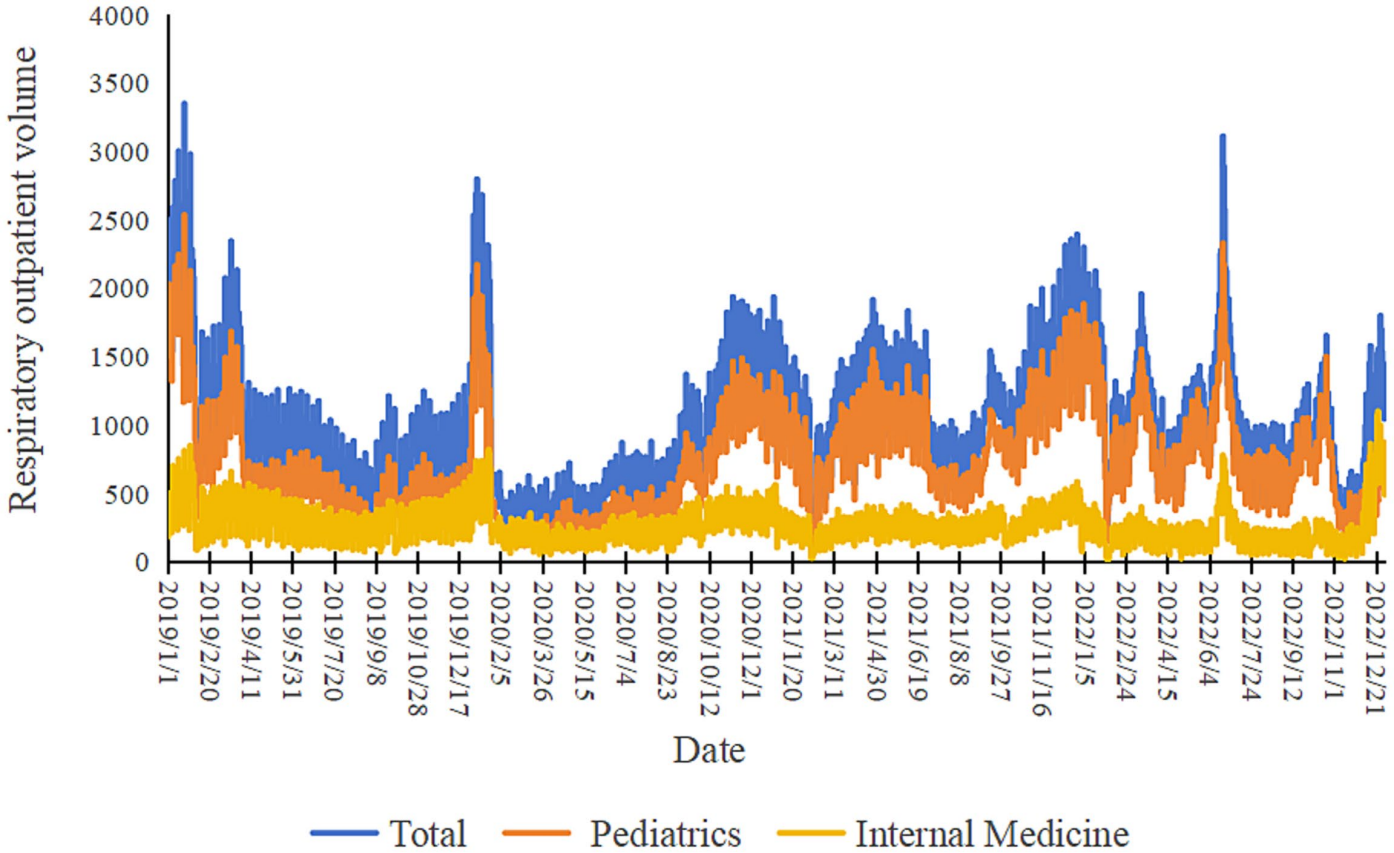
The trends in respiratory outpatient volumes, pediatric respiratory outpatient volumes and internal medicine respiratory outpatient volumes from 2019 to 2022.

Between January 2019 and December 2022, the total number of respiratory system disease outpatient visits across seven hospitals was 1,530,000. As shown in Table 1, the average daily outpatient visits for pediatric respiratory diseases were 758.58 ± 407.04, while the average daily outpatient visits for adult respiratory diseases in internal medicine were 288.65 ± 147.89. From Figure 1, it can be observed that there was a sharp decline in respiratory system outpatient visits at the beginning of 2020, followed by an overall annual increase in outpatient numbers. The outpatient visit numbers exhibited considerable fluctuations, with seasonal variations being more pronounced. In general, the winter and spring seasons saw higher outpatient visits compared to the summer and autumn seasons.

Among all hospitals, the most common respiratory disease by outpatient visits was acute upper respiratory tract infections, accounting for 25.60% of the total respiratory disease outpatient visits, with 391,607 visits. During this period, pediatric respiratory diseases accounted for a significant portion of the total respiratory outpatient visits in Fuzhou, representing 72.44% of the total, with acute upper respiratory tract infections, influenza and pneumonia, and other upper respiratory tract diseases being the predominant conditions, comprising 41.36, 27.55, and 13.06% of pediatric respiratory outpatient visits, respectively. For internal medicine respiratory diseases, the top three diseases by proportion in daily outpatient visits were acute upper respiratory infections (29.42%), other upper respiratory tract diseases (27.01%), and chronic lower respiratory diseases (26.47%), as shown in Table 3.

**Table 3:** The composition ratio (%) of the daily outpatient visits for respiratory outpatient visits in Fuzhou from 2019 to 2022.

Outpatients	Acute upper respiratory infection	Influenza and pneumonia	Other acute lower respiratory infections	Other upper respiratory tract diseases	Chronic lower respiratory diseases
Total	32.87%	9.87%	10.84%	24.06%	22.36%
Internal medicine	29.42%	7.99%	9.12%	27.01%	26.47%
Pediatrics	41.36%	27.55%	12.16%	13.06%	5.88%

### 3.3 Correlation analysis of air pollutants and meteorological factors with outpatient volume

**Figure 2:**
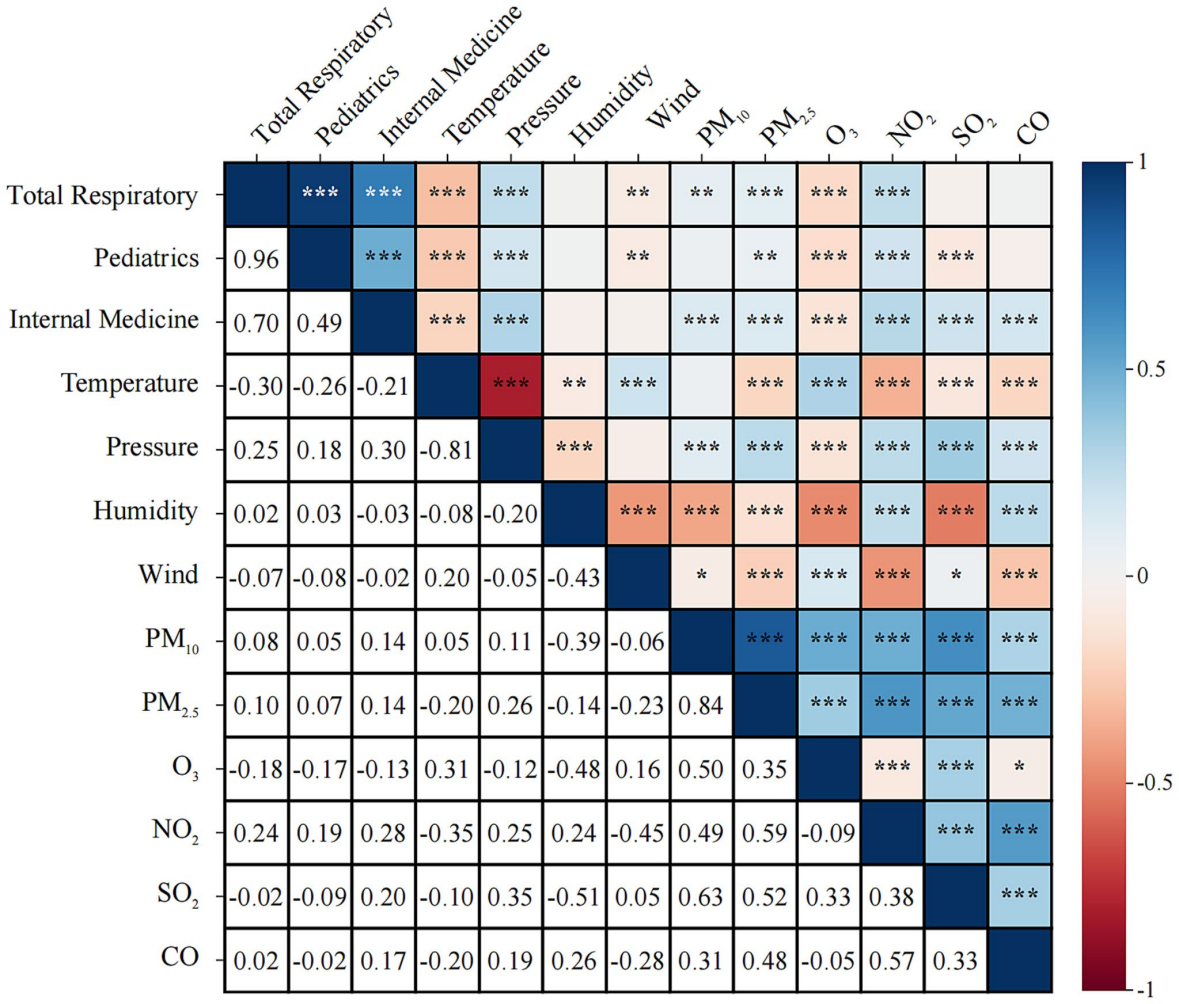
Analysis of the correlation between air pollutants, meteorological factors, and total outpatient volume and respiratory outpatient volume.

Air pollutants and meteorological factors were analyzed for correlation on respiratory outpatient visits. As shown in Figure 2, for overall respiratory disease visits, correlations existed for all except SO_2_, CO, and relative humidity. Positive correlations were found for all pollutants except O_3_; pediatric respiratory disease visits were negatively correlated with temperature, wind speed, O_3_, and SO_2_, and positively correlated with barometric pressure, PM_2.5_, and NO_2_; and internal medicine respiratory disease visits were negatively correlated with temperature and O_3_, and positively correlated with barometric pressure, PM₁₀, PM₂.₅, NO₂, SO₂, and CO. In the correlation analysis of meteorological factors and pollutants, humidity was negatively correlated with changes in the daily average concentrations of PM_2.5_, PM_10_, SO_2_, and O_3_, and temperature was negatively correlated with changes in the daily average concentrations of PM_2.5_, NO_2_, SO_2_, and CO. When the temperature decreased, the concentrations of PM_2.5_, NO_2_, SO_2_, and CO increased. Atmospheric pollutants and meteorological factors were included in the stepwise regression to analyze the influencing factors of respiratory disease outpatient volume, and the results, as shown in Table 4, showed that respiratory outpatient volume was significantly correlated only with Temperature, PM₂.₅, NO₂, SO₂, and PM₁₀ (*p* < 0.05), and the other factors were not statistically significant (*p* > 0.05).

**Table 4:** Analysis of the influence of atmospheric pollutants and meteorological factors on respiratory outpatient visits.

Variable	*β*	*t*	*p*
Temperature	−0.26	−6.95	0.000**
PM_2.5_	0.11	3.14	0.002**
NO_2_	0.39	10.8	0.000**
SO_2_	−0.36	−9.37	0.000**
PM_10_	−0.06	−1.98	0.048*
O_3_	0.04	0.37	0.72
CO	−0.07	−1.73	0.08
Atmospheric pressure (hPa)	−0.02	−0.05	0.87
Relative Humidity (%)	−0.008	−0.06	0.95
Humidity	−0.016	−0.123	0.93

*p < 0.05; **p < 0.01.

### 3.4 Impact of air pollutants on total outpatient volume and respiratory outpatient volume

**Figure 3:**
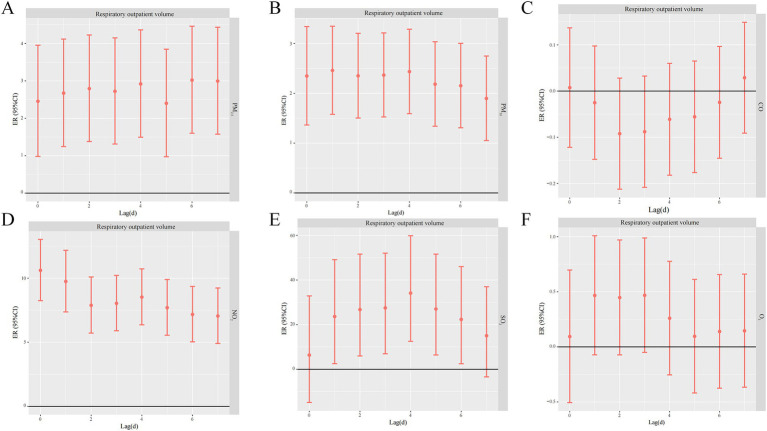
Lagged effects of different air pollutants on respiratory outpatient volume (**A** PM_2.5_, **B** PM_10_, **C** CO, **D** NO_2_, **E** SO_2_, **F** O_3_).

GLM model was constructed to analyze the effects of six air pollutants on the volume of respiratory outpatient visits in hospitals. As shown in Figure 3 and Supplementary Table S4, for the total respiratory outpatient visits, NO_2_ (ER: 10.61, 95%CI: 8.25–13.03) and PM_10_ (ER: 2.46, 95%CI: 1.58–3.35) reached the maximum in the lagged day 0–1 effect. SO_2_ (ER: 34.12, 95%CI: 12.53–59.87) peaked at lag day 4. PM_2.5_ (ER: 3.02, 95%CI: 1.06–4.46) was at lag day 6. However, the lags for CO and O_3_ were not statistically significant.

**Figure 4:**
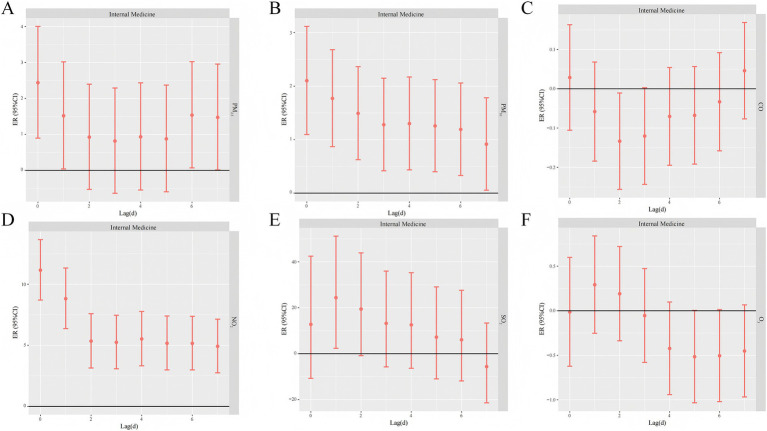
Lagged effects of different air pollutants on internal medicine respiratory outpatient volume (**A** PM_2.5_, **B** PM_10_, **C** CO, **D** NO_2_, **E** SO_2_, **F** O_3_).

PM_2.5_, PM_10_, NO_2_, and SO_2_ had differential effects on medical and pediatric respiratory visits, and all of them had significant effects and lagged risks of visits. As can be seen from the data in Supplementary Tables S5, S6, the effect of PM_2.5_ on pediatric lag4 respiratory disease visits (ER: 3.62, 95% CI: 2.06–5.20) was slightly higher than that on internal medicine lag0 (ER: 2.44, 95% CI: 0.90–4.00). The effect of PM_10_ on pediatric respiratory disease visits in lag1-4 The excess risk of lagged effect showed an increasing trend, with the highest ER value of 2.88% (95%CI: 1.95–3.82) at lag4; the most significant effect of PM_10_ on the volume of pediatric respiratory disease outpatient was found on lag0, with an ER value of 3.27% (95%CI: 1.76–4.80). The risk of NO_2_ on pediatric respiratory disease outpatient was slightly lower than that of medical respiratory disease outpatient clinics, with the highest ER values at lag0 for both clinics, with ER values of 10.61% (95%CI: 8.25–13.03) and 14.48% (95%CI: 10.86–18.21), respectively. The excess risk of lagged effects of SO_2_ on outpatient clinics for pediatric respiratory illnesses tended to be higher in lag1-4, with the highest ER value at lag4, reaching 42.10% (95%CI: 17.31–72.12). NO_2_ had a slightly lower risk for outpatient pediatric respiratory illnesses than medical respiratory illnesses. ER was highest at 42.10% (95%CI: 17.31–72.12), and the effect of SO_2_ on outpatient visits for medical respiratory diseases was highest at 24.39% (95%CI: 2.35–51.19) at lag1, and the risk of SO_2_ on outpatient visits for pediatric respiratory diseases was significantly higher than that for medical respiratory diseases (Figure 4).

**Figure 5:**
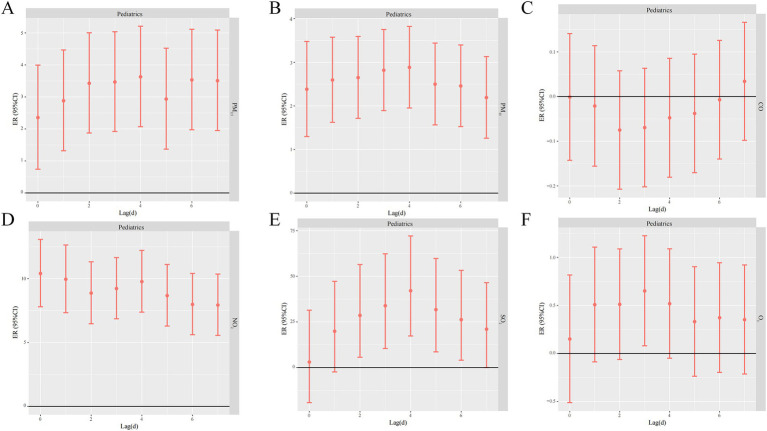
Lagged effects of different air pollutants on pediatric respiratory outpatient volume (**A** PM_2.5_, **B** PM_10_, **C** CO, **D** NO_2_, **E** SO_2_, **F** O_3_).

The lagged effect of O_3_ on pediatric respiratory disease visits was significant on day 3 with an ER of 0.65% (95% CI: 0.07–1.22). The one-day lagged effect of O_3_ on medical respiratory disease visits was not statistically significant at the 95% confidence interval. In contrast, there was no statistically significant one-day lagged effect of CO on the risk of daily respiratory disease visits in pediatrics and internal medicine (Figure 5).

### 3.5 Impact of air pollutants on total outpatient volume and respiratory outpatient volume

Supplementary Tables S7–S16 and Supplementary Figures S3–S12 show the correlation between each 10 μg/m^3^ of the concentrations of PM_2.5_, PM_10_, NO_2_, SO_2_, and O_3_ and daily outpatient visits for each type of respiratory diseases in pediatrics and internal medicine in urban areas of Fuzhou city, January 2019–December 2022, with the Lagged effect of outpatient visits. These pollutants had significant and lagged effects on the risk of outpatient visits for different subgroups of respiratory diseases in pediatrics. Since there was no statistically significant lagged effect of CO with all types of respiratory diseases in pediatrics and internal medicine in Fuzhou City, and no statistically significant lagged effect of O_3_ with all types of respiratory diseases in internal medicine, stratified analyses were not performed.

#### 3.5.1 Stratified analysis of the risk of daily outpatient visits for each type of respiratory diseases per 10 μg/m^3^ of pollutant elevation

Supplementary Tables S7–S11 and Supplementary Figures S3–S7 contain the daily outpatient visits of atmospheric pollutants (PM_2.5_, PM_10_, NO_2_, SO_2_, and O_3_) with various types of respiratory diseases of internal medicine in the urban area of Fuzhou City, Fuzhou City, China, from January 2019 to December 2022 Lagged effects. All these pollutants were significantly associated with the risk of outpatient visits for different subgroups of respiratory diseases in pediatrics.

(1) Stratified analysis of the risk of outpatient visits for various types of respiratory diseases in pediatrics per 10 μg/m^3^ increase in PM_2.5_ concentration:

PM_2.5_ had a significant and lagged effect on the risk of outpatient visits for different subgroups of respiratory diseases in pediatrics; the risk of outpatient visits for respiratory diseases for other acute lower respiratory tract infections in pediatrics was highest in lag2 (ER: 3.81, 95% CI: 2.09–5.55); outpatient risk for other diseases of the upper respiratory tract in pediatrics was highest at lag7 (ER: 2.37, 95% CI: 0.51–4.26), outpatient risk for respiratory diseases of chronic lower respiratory tract diseases in pediatrics was highest at lag6 (ER: 3.46, 95% CI: 1.71–5.25), and outpatient risk for respiratory diseases of influenza and pneumonia in pediatrics was highest at lag4 (ER: 5.39, 95% CI: 3.86–6.95) was the highest. The one-day lagged effect of daily pediatric acute upper respiratory tract infection visits was significant for the first 6 days and was highest with lag0 (ER: 2.46, 95% CI: 1.49–3.44).

(2) Stratified analysis of the risk of pediatric outpatient visits for various respiratory diseases per 10 μg/m^3^ increase in PM_10_ concentration:

PM_10_ had a significant effect on the risk of outpatient visits for different subgroups of respiratory diseases in pediatrics. The risk of respiratory diseases in pediatrics for acute upper respiratory tract infections was highest at lag0 (ER: 3.27, 95% CI: 1.76–4.80); the risk of respiratory diseases in pediatrics for other acute lower respiratory tract infections was highest at lag3 (ER: 3.05, 95% CI: 2.02–4.09); the risk of respiratory diseases in pediatrics for influenza and pneumonia were both highest at lag4 (ER: 3.05, 95% CI: 2.02–4.09); and the risk of respiratory diseases in pediatrics was highest at lag3 (ER: 3.05, 95% CI: 3.05–4.09). Risk was highest for lag4 (ER: 3.63, 95% CI: 2.71–4.57); lag6 was highest for other pediatric upper respiratory diseases and pediatric chronic lower respiratory diseases, and the effect of PM_10_ on pediatric chronic lower respiratory disease visits (ER: 2.39, 95% CI: 1.38–3.41) was slightly higher than that of other pediatric upper respiratory diseases (ER: 2.39, 95% CI: 1.38–3.41), diseases [ER: 1.72, 95%CI: (0.66–2.78)].

(3) Stratified analysis of the risk of pediatric outpatient visits for various respiratory diseases per 10 μg/m^3^ increase in NO_2_ concentration.

NO_2_ had a significant effect on the risk of outpatient visits for different subgroups of respiratory diseases with a lag. The effect of NO_2_ on the risk of outpatient visits for pediatric acute upper respiratory tract infections, and other diseases of the pediatric upper respiratory tract was greatest at lag0, with a greater effect on pediatric acute upper respiratory tract infection visits (ER: 11.62, 95%CI: 9.23–14.06), and a smaller effect on the risk of outpatient visits for other diseases of the pediatric upper respiratory tract (ER: 8.39, 95%CI: 5.41–11.45); NO_2_ had the highest effect on the risk of outpatient visits for respiratory diseases of other acute lower respiratory tract infections in pediatrics with lag6 (ER: 11.40, 95%CI: 8.68–14.19) was highest; the outpatient risk of respiratory disease for pediatric influenza and pneumonia was highest with lag5 (ER: 7.87, 95%CI: 5.47–10.33); and the outpatient risk of chronic lower respiratory disease in pediatrics was highest with lag1 (ER: 6.77, 95%CI: 3.91–9.71).

(4) Stratified analysis of the outpatient risk of various respiratory diseases in pediatrics per 10 μg/m^3^ increase in SO_2_ concentration.

SO_2_ had a significant effect on outpatient risk of different respiratory disease subgroups with a lag. lag1 (ER: 45.76, 95%CI: 20.38–76.48) had the greatest effect on outpatient risk of pediatrics acute upper respiratory infections. Risk impact on outpatient visits for pediatric influenza and pneumonia was greatest in lag4 (ER: 88.8, 95%CI: 52.63–133.55), outpatient visits for other pediatric acute lower respiratory infections was greatest in lag2 (ER: 71.06, 95%CI: 36.31–114.67), and outpatient visits for pediatric chronic lower respiratory diseases was greatest in lag5 (ER: 45.57, 95%CI: 17.59–80.2), and the one-day lagged effect of SO_2_ on the risk of pediatric outpatient visits for other upper respiratory tract diseases was not statistically significant at the 95% confidence interval.

(5) Stratified analysis of the risk of pediatric outpatient visits for various respiratory diseases per 0.1 mg/m^3^ increase in O_3_ concentration.

O_3_ had a significant effect on the risk of outpatient visits for different subgroups of respiratory diseases with lagged effects. lag1 had the greatest effect on the risk of outpatient visits for pediatric acute upper respiratory infections (ER: 0.68, 95% CI: 0.14–1.21); the impact of O_3_ on outpatient risk for pediatric influenza and pneumonia, pediatric other acute lower respiratory tract infections, and pediatric chronic lower respiratory tract diseases was all greatest with lag3, with the impact on outpatient volume for pediatric influenza and pneumonia (ER: 1.75, 95%CI: 1.14–2.37) being slightly greater than the impact on outpatient volume for other acute lower respiratory tract infections in pediatrics (ER: 1.00, 95%CI: 0.34–1.68); the risk effect of O_3_ on outpatient visits for other pediatric upper respiratory tract conditions had a single-day lagged effect versus a cumulative lagged effect that was not statistically significant at the 95% confidence interval.

#### 3.5.2 Stratified analysis of the risk of each 10 μg/m^3^ elevation of pollutants on the risk of outpatient visits for various types of respiratory diseases in internal medicine

Supplementary Tables S12–S16 and Supplementary Figures S8–S12 contain the lagged effects of each 10 μg/m^3^ elevation of the concentrations of PM_2.5_, PM_10_, NO_2_, and SO_2_ in the urban area of Fuzhou City from January 2019 to December 2022 on the daily outpatient visits for each type of respiratory diseases in internal medicine in Fuzhou City. The risks of these pollutants on outpatient visits for different subgroups of respiratory diseases in pediatrics were all correlated by significant correlations.

(1) Stratified analysis of the risk of daily outpatient visits for various types of respiratory diseases in internal medicine per 10 μg/m^3^ increase in PM_2.5_ concentration.

The risk of outpatient visits for other diseases of the upper respiratory tract in internal medicine and other acute lower respiratory tract infections in internal medicine were all highest at lag0, and the effect of PM_2.5_ on visits for other acute lower respiratory tract infections in internal medicine (ER: 5.21, 95%CI: 2.89–7.59) was slightly higher than that for other diseases of the upper respiratory tract in internal medicine (ER: 2.94, 95%CI: 0.38–5.57). The risk of outpatient visits for acute upper respiratory tract infections in internal medicine was highest with lag1 (ER: 3.13, 95%CI: 1.01–5.30), and the risk of outpatient visits for chronic lower respiratory tract diseases in internal medicine was highest with lag6 (ER: 1.82, 95%CI: 0.05–3.62). 95% confidence intervals were considered statistically non-significant for PM_2.5_ for influenza and pneumonia in internal medicine.

(2) Stratified analysis of the risk of daily outpatient visits for various respiratory diseases in internal medicine per 10 μg/m^3^ increase in PM_10_ concentration.

The risk of outpatient visits for other diseases of the upper respiratory tract in internal medicine and other acute lower respiratory tract infections in internal medicine was highest in lag0, and the effect of PM_10_ on visits for other acute lower respiratory tract infections in internal medicine (ER: 2.86, 95% CI: 1.41–4.33) was slightly higher than that of internal medicine other diseases of the upper respiratory tract (ER: 2.11, 95%CI: 0.56–3.69). The risk of outpatient visits for acute upper respiratory tract infections in internal medicine was highest with lag1 (ER: 3.13, 95%CI: 1.01–5.30), and the risk of outpatient visits for chronic lower respiratory tract diseases in internal medicine was highest with lag4 (ER: 1.39, 95%CI: 0.34–2.45). The lagged effect of PM_10_ on the daily outpatient risk of influenza and pneumonia was considered within 95% confidence intervals to be not statistically significant.

(3) Stratified analysis of the risk of daily outpatient visits for various respiratory diseases in internal medicine per 10 μg/m^3^ increase in NO_2_ concentration.

The effects of NO_2_ on the risk of outpatient visits for acute upper respiratory infections in internal medicine, other diseases of the upper respiratory tract in internal medicine, chronic lower respiratory diseases in internal medicine, influenza and pneumonia in internal medicine, and infections of other lower respiratory diseases in internal medicine were greatest at lag0, and the effect on the visit to the clinic for acute upper respiratory infections in internal medicine was the (ER: 14.48, 95%CI: 10.86–18.21) and to a lesser extent on the risk of outpatient visits for other acute lower respiratory tract infections in internal medicine (ER: 8.60, 95%CI: 3.13–14.35).

(4) Stratified analysis of the risk of daily outpatient visits for various types of respiratory diseases in internal medicine per 10 μg/m^3^ increase in SO_2_ concentration.

The risk of outpatient visits for acute upper respiratory tract infections in internal medicine was highest with lag1 (ER: 52.00, 95%CI: 14.95–101.00), the risk of outpatient visits for influenza and pneumonia in internal medicine was highest with lag6 (ER: 57.36, 95%CI: 2.07–142.62), and the risk of outpatient visits for other acute lower respiratory tract infections in internal medicine was highest with lag2 (ER: 31.87, 95%CI: 0.58–72.91; 142.62), outpatient risk of other acute lower respiratory tract infections in internal medicine was highest with lag2 (ER: 31.87, 95%CI: 0.58–72.91), outpatient risk of other diseases of the upper respiratory tract in internal medicine was highest with lag4 (ER: 42.10, 95%CI: 17.31–72.12), outpatient risk of chronic lower respiratory diseases in internal medicine was highest with lag4 (ER: 42.10, 95%CI: 17.31–72.12), and outpatient risk of chronic lower respiratory diseases in internal medicine outpatient risk was highest with lag3 (ER: 27.33, 95%CI: 2.17–58.67).

### 3.6 Sensitivity analysis

Supplementary Figures S13–S15 show the sensitivity value analysis of modifying the df values of the covariates, from which it can be seen that the results of the analysis after modifying the df are generally similar to the original results. Therefore, overall the lag model is more stable.

## 4 Discussion

In this study, the relationship between air pollutants and respiratory diseases in Fuzhou City during the period from January 2019 to December 2022 was comprehensively analyzed. The results of the study showed that air pollution, especially PM_2.5_, PM_10_, NO_2_, and SO_2,_ significantly and usually with a lagged effect affected respiratory outpatient clinic visits, especially in the pediatric and adult populations. The study used air quality, meteorological factors, and detailed outpatient records from seven hospitals in Fuzhou City, which provide a solid basis for examining the public health impacts of air pollution.

### 4.1 Impact of air pollutants and meteorological factors on outpatient visits for respiratory diseases

During the period from January 2019 to December 2022, air pollutants in Fuzhou City showed an overall decreasing trend. This is due to the fact that the Chinese government attaches great importance to the prevention and control of air pollution and continues to promote the improvement of atmospheric environmental quality, and the number of heavily polluted days across the country continues to decrease (26). According to China’s secondary air quality standards, only PM_2.5_ and O_3_-8h have exceedances, but the exceedance rates are only 0.16 and 1.7%. Thus, this indicates that air pollution prevention and control in Fuzhou City has been effective. Nonetheless, the number of respiratory outpatient visits in general still showed an increasing trend year by year. This may be related to meteorological factors (e.g., temperature) and increased demand for medical services in the context of the new Crown pneumonia epidemic (27, 28).

Changes in daily outpatient visits for respiratory diseases during the study period were characterized by high rates in winter and spring and low rates in summer and fall. As a result of the onset of the COVID-19 pandemic (29), a series of public health interventions such as social distancing, wearing masks, and other isolation methods by governmental organizations resulted in the lowest respiratory outpatient visits in hospitals in the early 2020s, with a particularly significant impact on children. Total respiratory outpatient visits were lower after the outbreak than before, and there was a brief burst of respiratory outpatient visits. The trends in daily mean fluctuations of PM_2.5_ and PM_10_ were similar, with the highest peaks occurring mostly in winter and spring.

The results of the time series analysis of air pollutants and hospital outpatient visits showed that increasing pollutant concentrations increased the risk of respiratory diseases. In respiratory outpatient visits, lagged effects were observed for all pollutants except CO. Our results are consistent with several epidemiologic studies demonstrating that air pollutants affect the human respiratory system (30, 31). A study in Baotou, China, similar to the results of this study (32), observed that there was a significant association between pollutant concentrations and respiratory outpatient visits.

Temperature and atmospheric pressure are key meteorological factors that influence the association between air pollution and hospital visits. Our results demonstrate that exposure conditions to pollutant concentrations at lower temperatures and higher barometric pressures increase the risk of respiratory diseases, which may be related to the reduced diffusion capacity of pollutants at low temperatures and high pressures, as well as the weakening of the body’s respiratory defense mechanisms. Some studies have confirmed that low temperature conditions increase the risk of respiratory diseases. While low-temperature conditions impede the dispersion of pollutants, high-pressure environments may limit the vertical dispersion of pollutants, resulting in higher concentrations of particulate matter and harmful gases (33). Some studies have confirmed that low temperature conditions increase the risk of respiratory diseases (34, 35). While low-temperature conditions impede the diffusion of pollutants, high humidity may increase the viscosity of the respiratory mucosa, making it less capable of clearing foreign bodies (36). There is consistency with several previous studies (37–39). Therefore, we recommend taking protective measures such as wearing masks when going out in winter and spring to reduce the risk of respiratory diseases.

### 4.2 Lagged effects of atmospheric pollutants on the risk of respiratory disease outpatient visits in Fuzhou City

In the study of time series data on respiratory system diseases in Fuzhou City and various air pollutants, the maximum single-day lag effect of PM_2.5_ occurred on the 6th day. A study in Utah (40) found that short-term elevations in PM_2.5_ concentrations caused an increase in the incidence of acute respiratory infections in a large number of patients, suggesting that PM_2.5_ levels may influence the severity of acute respiratory infections. In contrast, a study by a European multicenter group showed that the relationship between PM_2.5_ and the number of outpatient visits for respiratory diseases was not significant (41). This is in contrast to our results, which showed that a 10 mg/m^3^ increase in PM_2.5_ was associated with an excess risk of respiratory disease outpatient clinic visits at lag6 of ER 3.02% (95% CI: 1.06–4.46), and that these differences were due to a number of factors. First, in our study, the study population was residents of Fuzhou City, which is a mild pollution concentration area, while Karakatsani et al. were concerned mainly with high pollution areas, and according to J Schwartz’s study (42) it is known that daily outpatient visits for respiratory illnesses change more drastically when they increase at low levels of air pollutant concentration, while the concentration of air pollutants increases significantly, the number of daily outpatient visits for respiratory diseases tends to stabilize or even decrease, the increase in daily visits for respiratory illnesses leveled off or even declined at high levels of air pollutant concentrations. Other possible reasons include differences in meteorological factors, sources of air pollutants, levels of air pollutants, or chemical composition in different study areas.

The effect of each 10 μg/m^3^ increase in PM_10_ concentration on the total number of respiratory disease outpatient visits reached a maximum on day 1, with an ER of 2.46% (95% CI: 1.58–3.35), and the risk of increased respiratory disease outpatient visits appeared earlier than that of PM_2.5_, which may be related to the mechanism of the action of airborne particulate matter on the respiratory system, and the sources, compositions, and sites of deposition in the body are different between PM_2.5_ and PM_10_. The sources, composition, and deposition sites within the body of PM_2.5_ and PM_10_ are different, PM_10_ aerodynamic diameter is larger, usually deposited in the upper respiratory tract, bronchial or fine bronchial tubes, caused by respiratory disease symptoms appear earlier, while PM_2.5_ aerodynamic diameter is smaller, can be deep into the alveoli.

The lagged effects of NO_2_ were all greatest on the daily outpatient visits for respiratory diseases on the same day, and NO_2_ can rapidly stimulate respiratory diseases in humans. Among all the pollutants considered in this study, all pollutants (PM_10_, PM_2.5_, SO_2_, NO_2_, and O_3_-8h) had lagged effects on the total number of outpatient visits for respiratory diseases, with the exception of CO, and the short-term exposures to CO had lagged effects on the total number of outpatient visits for respiratory diseases in the same day with the daily respiratory disease visits did not show significant effects for all lag periods, suggesting that it has a small or delayed effect on respiratory visits. The failure to produce significant results for these pollutants may reflect the relatively transient nature of their health effects or the fact that specific meteorological conditions during the study period diminished their effects on respiratory health (43). Although the link between CO and cardiovascular disease risk is well established (44), its role on the respiratory system has not been confirmed, side by side demonstrating the validity of the data used in this study. Therefore, the role of CO in respiratory diseases needs to be further investigated.

### 4.3 Heterogeneity of lagged effects of atmospheric pollutants and respiratory diseases

In this study, respiratory outpatient clinics were further divided into pediatric and internal medicine types of respiratory diseases with daily mean concentrations of atmospheric pollutants for time series analysis in order to explore the different effects of each pollutant on the volume of pediatric and internal medicine outpatient clinics for each respiratory disease. It was found that acute infections of the upper respiratory tract in both pediatrics and internal medicine had the greatest impact on the risk of outpatient visits on the day of pollution or on the first day after pollution, which shows that acute infections of the upper respiratory tract are very sensitive to air pollution.

PM_2.5_ and PM_10_ had the highest outpatient risk of upper respiratory tract acute infections in internal medicine at lag1, while the outpatient risk of upper respiratory tract acute infections in pediatrics was highest at lag0, which may be due to the fact that the upper respiratory tracts of pediatric patients are more susceptible to direct stimulation by air pollutants than those of adults, which leads to a rapid onset of acute inflammatory responses.

In this study, it was found that all types of respiratory diseases in pediatrics, except for other diseases of the upper respiratory tract in pediatrics, were more affected by the increased concentration of SO_2_ pollution, and the ER value of SO_2_ on the risk of daily outpatient visits for influenza and pneumonia in pediatrics was as high as 88.80% (95%CI: 52.63–133.55); all types of respiratory diseases in internal medicine were more affected by the increased concentration of SO_2_ pollution, and SO_2_ was also a major contributor to the risk of daily outpatient visits for infections in internal medicine. SO_2_ also had the greatest single-day lagged effect on the risk of daily outpatient visits for influenza and pneumonia in internal medicine, with ER values as high as 57.36% (95%CI: 2.07–142.62). This may be related to the mechanism of action of SO_2_, as SO_2_ is soluble in water, and after entering the respiratory tract, it can combine with the moisture on the respiratory mucosa to form corrosive nitrite, sulfuric acid, etc., with strong pathogenicity (45).

It was found that elevated NO_2_ concentration could cause elevated risk of increased daily outpatient visits for various types of pediatric respiratory diseases, and daily outpatient visits for acute upper respiratory tract infections were more affected by atmospheric NO_2_ concentration, and the excess risk could reach the maximum value in lag0, with an ER value of 11.62% (95% CI: 9.23–14.06), which is in agreement with the findings of Liu et al. (46) The single-day lag effects of daily outpatient visits for pediatric upper respiratory diseases were all higher in terms of the excess risk from the day of the rise in NO_2_ concentration to the second day, while the single-day lag effects of lower respiratory diseases were mostly higher in terms of the excess risk from the fifth to the sixth day of the rise in NO_2_ concentration, probably because upper respiratory infections are mainly in the nasal cavity area, pharynx, and larynx, which are prone to acute inflammation, commonly seen in common colds and tonsillitis, pharyngitis and other diseases with faster onset, while lower respiratory infections are mainly in the trachea, bronchus, lungs, interstitium and other parts of the lungs, commonly seen in bronchitis, bronchiolitis, pneumonia and other diseases with slower onset. For all types of pediatric respiratory diseases, the effect of elevated NO_2_ concentrations is maximized on the day of lag. This may be due to the fact that NO₂ is a strong oxidizing agent capable of directly damaging respiratory epithelial cells and triggering an inflammatory response (47). In addition, NO₂ has a high chemical reactivity in the atmosphere and tends to synergize with other pollutants (e.g., particulate matter), further exacerbating respiratory irritation (48).

With a large sample size and strict quality control, this study reveals the relationship between exposure to low-level air pollutants and hospital respiratory outpatient visits, which is highly scientific and representative. However, this study also has some limitations. First, the results of the study may be biased due to the limited nature of the outpatient volume data and the failure to control for confounding factors that may affect the results of the study. The seven hospitals selected for this study were all representative of the urban areas of Fuzhou City with strong comprehensive strength, but due to limitations in data acquisition, each hospital was only able to provide respiratory outpatient data for internal medicine and pediatrics, lacking key demographic information such as age, gender, and address. In addition, factors such as socioeconomic status and vaccination rates may influence an individual’s sensitivity to air pollution as well as his or her healthcare-seeking behavior, but these factors could not be adequately considered in this study. Regarding the exposure to air pollutants, this study used the average concentrations at national monitoring sites, which could not accurately reflect the actual exposure levels of individuals. Meanwhile, there may be a correlation between indoor air pollution and outdoor air pollution, but their independent effects may not be fully captured by the model in this study (49–50). Therefore, future studies should further develop more comprehensive time-series analyses of respiratory disease data, collect more information at the individual and group levels, and provide more scientific epidemiological support for the development of policies and measures to protect respiratory health in areas with low pollutant exposures by means of data collection and analysis from multiple perspectives.

## 5 Conclusion

In summary, this study confirms that changes in the concentrations of PM_2.5_, PM_10_, NO_2_ and SO_2_ have a significant effect on the number of outpatient visits for respiratory diseases in Fuzhou City. The effects of pollutants on respiratory diseases were not only immediate but also showed lagged effects, with NO_2_ and PM_10_ pollutants having faster effects and SO_2_ and PM_2.5_ having longer lagged effects, especially in pediatric outpatient cases. Overall, the excess risk of elevated pollutants on pediatric respiratory disease clinics was higher than that of medical respiratory disease clinics, and despite the relatively good air quality in Fuzhou City, the results of the study suggest that air pollution in low-pollution areas still has a significant impact on public health, especially on susceptible groups such as children. Therefore, the development of appropriate air pollution control policies, especially in low-pollution areas, remains key to safeguarding public health. This study provides an important epidemiologic basis for air pollution control and respiratory disease management in low-pollution areas.

## Data Availability

The raw data supporting the conclusions of this article will be made available by the authors, without undue reservation.
