# Supplementary material for: Time series analysis of low-concentration air pollution and hospital respiratory disease outpatient visits
Source: Front Public Health. 2025 May 20;13:1585086. doi: 10.3389/fpubh.2025.1585086 (PMC12129957; doi:10.3389/fpubh.2025.1585086)
Supplement: Supplementary file 1 [file Data_Sheet_1.docx]

**Table S1 ARIMA(1,1,2) Model Parameter Table**

| Model | RMSE | MSE | MAE | MAPE | ACF1 | Ljung-Box | |
| --- | --- | --- | --- | --- | --- | --- | --- |
|  |  |  |  |  |  | Q-statistic(Q2) | p-value |
| ARIMA(1,1,2) | 256.1239 | 65599.45 | 187.2514 | 0.2205 | 0.69 | 18.43 | 0.0082 |

**Table S2 The trend of changes(Mean and Standard Deviation) of air pollutants and meteorological factors in Fuzhou from 2019 to 2022**

| Variables | 2019  Mean±SD | 2020  Mean±SD | 2021  Mean±SD | 2022  Mean±SD |
| --- | --- | --- | --- | --- |
| PM_10_ (μg/m^3^) | 41.78 ± 18.400 | 37.64 ± 15.845 | 38.28 ± 15.867 | 30.77 ± 13.566 |
| PM_2.5_ ( μg/m^3^) | 22.70 ± 11.152 | 19.21 ± 8.948 | 19.12 ± 8.881 | 16.86 ± 9.205 |
| NO_2_ (μg/m^3^) | 21.11 ± 9.027 | 19.42 ± 7.650 | 18.49 ± 7.962 | 13.42 ± 5.588 |
| SO_2_ (μg/m^3^) | 4.45 ± 0.860 | 4.41 ± 0.745 | 3.70 ± 0.884 | 3.22 ± 0.787 |
| CO (mg/m^3^) | 0.65 ± 0.135 | 0.65 ± 0.116 | 0.61 ± 0.110 | 0.45 ± 0.118 |
| O_3_-8h (μg/m^3^) | 91.48 ± 32.903 | 90.55 ± 28.672 | 84.55 ± 24.808 | 94.44 ± 30.154 |
| Atmospheric pressure (hPa) | 1011.55 ± 7.909 | 1005.46 ± 7.072 | 1004.51 ± 7.672 | 1002.89(21.681 |
| Temperature ( °C) | 21.68 ± 6.514 | 21.48 ± 6.870 | 21.79 ± 6.753 | 21.25 ± 7.387 |
| Humidity (%) | 72.85 ± 10.828 | 72.95 ± 12.980 | 74.48 ± 13.420 | 72.92 ± 14.254 |

**Table S3 Concentrations of six major air pollutants at different air quality levels**

**Chinese Concentration limits ^a^**

| **Pollutants** | **Average time** | **Class Ⅰ** | **Class Ⅱ** | **WHO’s limits** |
| --- | --- | --- | --- | --- |
| PM2.5(μg/m^3^) | Annual mean  Per 24h | 15  35 | 35  75 | 10  25 |
| PM10(μg/m^3^) | Annual mean  Per 24h | 40  50 | 70  150 | 20  50 |
| NO2(μg/m^3^) | Annual mean  Per 24h | 40  80 | 40  80 | 40  - |
| SO2(μg/m^3^) | Annual mean  Per 24h | 20  50 | 60  150 | -  20 |
| O3(μg/m^3^) | Maximum 8h | 100 | 160 | 100 |
| CO(mg/m^3^） | Per 24h | 4 | 4 | - |

**Table S4** The lag effect of each 10 μg/m^3^ (0.1 mg/m^3^ for O3) increase in pollutants concentration on the number of respiratory outpatient volume

| Lagging days | PM_10_ | PM_2.5_ | NO_2_ | SO_2_ | CO | O_3_ |
| --- | --- | --- | --- | --- | --- | --- |
|  | ER (95%CI) | ER (95%CI) | ER (95%CI) | ER (95%CI) | ER (95%CI) | ER (95%CI) |
| lag0 | 2.35(1.36-3.34) | 2.46(0.98-3.95) | 10.61(8.25-13.03) | 6.36(-14.87-32.88) | 0.01(-0.12-0.14) | 0.09(-0.51-0.70) |
| lag1 | 2.46(1.58-3.35) | 2.67(1.24-4.12) | 9.75(7.37-12.18) | 23.64(2.51-49.13) | -0.03(-0.15-0.1) | 0.47(-0.07-1.01) |
| lag2 | 2.35(1.50-3.21) | 2.79(1.38-4.23) | 7.88(5.72-10.10) | 26.75(5.97-51.60) | -0.09(-0.21-0.03) | 0.45(-0.07-0.97) |
| lag3 | 2.37(1.53-3.21) | 2.72(1.31-4.15) | 8.04(5.90-10.22) | 27.51(6.94-52.04) | -0.09(-0.21-0.03) | 0.47(-0.05-0.99) |
| lag4 | 2.44(1.59-3.29) | 2.92(1.49-4.36) | 8.53(6.36-10.73) | 34.12(12.53-59.87) | -0.06(-0.18-0.06) | 0.26(-0.25-0.78) |
| lag5 | 2.18(1.34-3.04) | 2.40(0.97-3.85) | 7.70(5.55-9.90) | 27.01(6.41-51.60) | -0.06(-0.18-0.07) | 0.10(-0.42-0.61) |
| lag6 | 2.15(1.31-3.00) | 3.02(1.06-4.46) | 7.17(5.03-9.36) | 22.34(2.48-46.04) | -0.02(-0.15-0.1) | 0.14(-0.37-0.66) |
| lag7 | 1.90(1.05-2.75) | 2.99(1.57-4.43) | 7.05(4.91-9.24) | 15.04(-3.40-37.00) | 0.03(-0.09-0.15) | 0.15(-0.37-0.66) |

**Table S5** The lag effect of each 10 μg/m^3^ (0.1 mg/m^3^ for O3) increase in pollutants concentration on the number of internal medicine respiratory outpatient volume

| Lagging days | PM_10_  ER (95%CI) | PM_2.5_  ER (95%CI) | NO2  ER (95%CI) | SO2  ER (95%CI) | CO  ER (95%CI) | O3  ER (95%CI) |
| --- | --- | --- | --- | --- | --- | --- |
| lag0 | 2.10(1.10-3.12) | 2.44(1.01-4.00) | 14.48(10.86-18.21) | 12.79(-10.69-42.45) | 0.03(-0.11-0.16) | -0.01(-0.62-0.6) |
| lag1 | 1.77(0.87-2.68) | 1.52(0.04-3.02) | 11.83(8.23-15.55) | 24.39(2.35-51.19) | -0.06(-0.18-0.07) | 0.29(-0.25-0.84) |
| lag2 | 1.49(0.63-2.36) | 0.92(-0.53-2.4) | 7.11(3.91-10.40) | 19.43(-0.85-43.89) | -0.13(-0.26--0.01) | 0.19(-0.34-0.72) |
| lag3 | 1.28(0.42-2.15) | 0.82(-0.64-2.29) | 8.06(4.88-11.34) | 13.21(-5.73-35.96) | -0.12(-0.24-0) | -0.05(-0.58-0.47) |
| lag4 | 1.3(0.43-2.17) | 0.93(-0.55-2.43) | 7.62(4.41-10.93) | 12.56(-6.32-35.26) | -0.07(-0.19-0.05) | -0.42(-0.94-0.1) |
| lag5 | 1.26(0.40-2.12) | 0.88(-0.59-2.37) | 7.29(4.09-10.58) | 7.21(-10.95-29.09) | -0.07(-0.19-0.06) | -0.52(-1.03-0) |
| lag6 | 1.19(0.33-2.06) | 1.54(0.07-3.02) | 8.59(5.40-11.88) | 6.06(-11.84-27.6) | -0.03(-0.16-0.09) | -0.51(-1.02-0.01) |
| lag7 | 0.92(0.05-1.78) | 1.47(0.01-2.96) | 7.08(3.90-10.35) | -5.6(-21.38-13.34) | 0.05（-0.08-0.17） | -0.45(-0.97-0.07) |

**Table S6** The lag effect of each 10 μg/m^3^ (0.1 mg/m^3^ for O_3_) increase in pollutants concentration on the number of predicate respiratory outpatient volume

| Lagging days | PM_10_  ER (95%CI) | PM_2.5_  ER (95%CI) | NO2  ER (95%CI) | SO2  ER (95%CI) | CO  ER (95%CI) | O3  ER (95%CI) |
| --- | --- | --- | --- | --- | --- | --- |
| lag0 | 2.38(1.29-3.47) | 2.35(0.74-3.99) | 10.39(7.79-13.06) | 3.04(-19.2-31.41) | 0.00(-0.14-0.14) | 0.15(-0.51-0.81) |
| lag1 | 2.59((1.62-3.57) | 2.88(1.31-4.46) | 9.94(7.32-12.62) | 19.89(-2.37-47.24) | -0.02(-0.15-0.11) | 0.5(-0.08-1.1) |
| lag2 | 2.64(1.71-3.59) | 3.42(1.87-5.00) | 8.86(6.46-11.31) | 28.57(5.66-56.46) | -0.07(-0.2-0.05) | 0.51(-0.06-1.08) |
| lag3 | 2.81(1.89-3.75) | 3.46(1.91-5.03) | 9.22(6.85-11.64) | 33.89(10.45-62.32) | -0.06(-0.2-0.06) | 0.65(0.07-1.22) |
| lag4 | 2.88(1.95-3.82) | 3.62(2.06-5.20) | 9.76(7.37-12.20) | 42.10(17.31-72.12) | -0.04(-0.18-0.08) | 0.51(-0.04-1.08) |
| lag5 | 2.49(1.56-3.44) | 2.93(1.36-4.52) | 8.66(6.28-11.10) | 31.72(8.6-59.78) | -0.03(-0.17-0.09) | 0.33(-0.23-0.9) |
| lag6 | 2.45(1.52-3.39) | 3.53(1.97-5.11) | 7.97(5.60-10.40) | 26.28(4.05-53.26) | 0.00(-0.13-0.12) | 0.37(-0.19-0.94) |
| lag7 | 2.19(1.25-3.13) | 3.50(1.94-5.08) | 7.92(5.55-10.35) | 21.06(0.02-46.52) | 0.03(-0.09-0.16) | 0.35(-0.21-0.92) |

**Table S7** The lag effect of each 10 μg/m^3^ (0.1 mg/m^3^ for O_3_) increase in pollutants concentration on the number of predicate acute upper respiratory infection outpatient volume

| Lagging days | PM_10_  ER (95%CI) | PM_2.5_  ER (95%CI) | NO2  ER (95%CI) | SO2  ER (95%CI) | CO  ER (95%CI) | O3  ER (95%CI) |
| --- | --- | --- | --- | --- | --- | --- |
| lag0 | 3.27(1.76-4.80) | 2.46(1.49-3.44) | 11.62(9.23-14.06) | 31.27(4.83-64.36) | 0.07(-0.06-0.20) | 0.48(-0.11-1.08) |
| lag1 | 2.94(1.49-4.42) | 2.45(1.58-3.33) | 10.41(8.03-12.84) | 45.76(20.38-76.48) | 0.01(-0.12-0.13) | 0.68(0.14-1.21) |
| lag2 | 2.19(0.75-3.64) | 1.93(1.09-2.77) | 7.38(5.20-9.60) | 41.46(17.76-69.93) | -0.06(-0.18-0.06) | 0.66(0.15-1.17) |
| lag3 | 2.08(0.65-3.54) | 1.84(1.00-2.68) | 8.49(6.34-10.69) | 35.32(12.9-62.20) | -0.02(-0.14-0.1) | 0.43(-0.08-0.94) |
| lag4 | 2.07(0.63-3.53) | 1.74(0.91-2.59) | 9.06(6.88-11.28) | 32.06(10.25-58.19) | 0.03(-0.09-0.15) | 0.13(-0.37-0.64) |
| lag5 | 0.80(-0.64-2.26) | 1.04(0.20-1.88) | 8.24(6.07-10.45) | 13.97(-4.98-36.7) | 0.02(-0.10-0.14) | -0.18(-0.68-0.33) |
| lag6 | 1.26(-0.17-2.72) | 1.03(0.20-1.87) | 8.28(6.13-10.48) | 10.54(-7.90-32.66) | 0.09(-0.03-0.21) | -0.33(-0.83-0.18) |
| lag7 | 1.12(-0.32-2.57) | 0.61(-0.23-1.45) | 7.60(5.45-9.80) | 0.73(-15.87-20.61) | 0.10(-0.02-0.22) | -0.37(-0.87-0.14) |

**Table S8** The lag effect of each 10 μg/m^3^ (0.1 mg/m^3^ for O_3_) increase in pollutants concentration on the number of pediatric influenza and pneumonia outpatient volume

| Lagging days | PM_10_  ER (95%CI) | PM_2.5_  ER (95%CI) | NO2  ER (95%CI) | SO2  ER (95%CI) | CO  ER (95%CI) | O3  ER (95%CI) |
| --- | --- | --- | --- | --- | --- | --- |
| lag0 | 2.05(0.94-3.17) | 3.66(2.03-5.32) | 4.04(1.45-6.69) | -5.16(-27.70-24.40) | 0.12(-0.02-0.26) | 0.29(-0.44-1.02) |
| lag1 | 2.63(1.65-3.62) | 4.49(2.94-6.06) | 4.96(2.37-7.60) | 35.51(7.57-70.70) | 0.11(-0.03-0.24) | 1.14(0.49-1.79) |
| lag2 | 2.95(2.01-3.89) | 4.11(2.58-5.66) | 4.66(2.27-7.11) | 50.73(21.22-87.41) | 0.03(-0.09-0.16) | 0.82(0.20-1.43) |
| lag3 | 3.35(2.43-4.28) | 4.82(3.29-6.37) | 3.81(1.48-6.21) | 85.76(49.89-130.23) | 0.01(-0.12-0.14) | 1.75(1.14-2.37) |
| lag4 | 3.63(2.71-4.57) | 5.39(3.86-6.95) | 6.85(4.44-9.32) | 88.8(52.63-133.55) | 0.10(-0.03-0.23) | 1.42(0.82-2.03) |
| lag5 | 3.19(2.27-4.11) | 4.85(3.32-6.40) | 7.87(5.47-10.33) | 85.53(50.20-129.18) | 0.14(0.01-0.27) | 1.05(0.45-1.66) |
| lag6 | 2.86(1.94-3.79) | 4.46(2.92-6.01) | 6.36(3.98-8.80) | 60.2(29.37-98.38) | 0.10(-0.02-0.23) | 1.41(0.81-2.02) |
| lag7 | 2.57(1.64-3.50) | 4.33(2.80-5.89) | 6.76(4.38-9.19) | 52.9(23.76-88.90) | 0.17(0.05-0.30) | 1.40(0.80-2.00) |

**Table S9** The lag effect of each 10 μg/m^3^ (0.1 mg/m^3^ for O_3_) increase in pollutants concentration on the number of pediatric other acute lower respiratory Infections outpatient volume

| Lagging days | PM_10_  ER (95%CI) | PM_2.5_  ER (95%CI) | NO2  ER (95%CI) | SO2  ER (95%CI) | CO  ER (95%CI) | O3  ER (95%CI) |
| --- | --- | --- | --- | --- | --- | --- |
| lag0 | 1.90(0.70-3.11) | 2.29(0.50-4.10) | 7.30(4.42-10.25) | 6.62(-19.42-41.09) | -0.08(-0.24-0.07) | 0.09(-0.69-0.88) |
| lag1 | 2.42(1.35-3.50) | 3.24(1.51-4.99) | 7.92(5.00-10.93) | 40.47(10.61-78.40) | -0.02(-0.17-0.13) | 0.60(-0.10-1.03) |
| lag2 | 2.98(1.96-4.02) | 3.81(2.09-5.55) | 8.43(5.73-11.20) | 71.06(36.31-114.67) | -0.01(-0.16-0.14) | 0.80(0.13-1.47) |
| lag3 | 3.05(2.02-4.09) | 3.61(1.90-5.36) | 7.98(5.32-10.70) | 66.68(33.01-108.89) | -0.02(-0.16-0.13) | 1.00(0.34-1.68) |
| lag4 | 2.60(1.57-3.65) | 2.92(1.20-4.67) | 8.75(6.06-11.52) | 57.69(25.80-97.68) | 0.07(-0.08-0.21) | 0.74(0.08-1.41) |
| lag5 | 2.82(1.80-3.85) | 3.20(1.48-4.96) | 10.55(7.83-13.34) | 45.42(15.94-82.41) | 0.04(-0.10-0.19) | 0.63(-0.03-1.29) |
| lag6 | 2.83(1.81-3.86) | 3.67(1.95-5.42) | 11.40(8.68-14.19) | 57.38(25.61-97.18) | 0.09(-0.06-0.23) | 0.70(0.04-1.37) |
| lag7 | 2.32(1.29-3.36) | 3.04(1.33-4.79) | 10.22(7.52-12.98) | 51.11(20.92-88.83) | 0.12(-0.02-0.27) | 0.77(0.11-1.44) |

**Table S10** The lag effect of each 10 μg/m^3^ (0.1 mg/m^3^ for O_3_) increase in pollutants concentration on the number of pediatric other upper respiratory tract diseases outpatient volume

| Lagging days | PM_10_  ER (95%CI) | PM_2.5_  ER (95%CI) | NO2  ER (95%CI) | SO2  ER (95%CI) | CO  ER (95%CI) | O3  ER (95%CI) |
| --- | --- | --- | --- | --- | --- | --- |
| lag0 | 1.69(0.47-2.92) | 1.58(-0.36-3.56) | 8.39(5.41-11.45) | 2.31(-22.47-35.02) | 0.14(-0.03-0.30) | 0.45(-0.25-1.15) |
| lag1 | 1.17(0.06-2.29) | 0.97(-0.92-2.89) | 7.70(4.7-10.79) | 0.11(-20.83-26.59) | 0.08(-0.08-0.24) | 0.60(-0.04-1.24) |
| lag2 | 1.51(0.44-2.59) | 1.84(-0.04-3.75) | 7.01(4.24-9.86) | 10.02(-12.15-37.78) | 0.02(-0.14-0.17) | 0.41(-0.21-1.04) |
| lag3 | 1.31(0.26-2.38) | 1.16(-0.68-3.04) | 5.66(2.97-8.41) | 3.35(-17.13-28.89) | -0.1(-0.26-0.05) | 0.52(-0.10-1.15) |
| lag4 | 1.11(0.04-2.18) | 0.72(-1.15-2.62) | 4.54(1.86-7.29) | 19.20(-4.20-48.31) | -0.07(-0.22-0.09) | 0.24(-0.37-0.86) |
| lag5 | 1.40(0.34-2.46) | 0.85(-1.02-2.76) | 3.83(1.15-6.59) | 8.82(-12.78-35.78) | -0.03(-0.19-0.12) | -0.05(-0.66-0.57) |
| lag6 | 1.72(0.66-2.78) | 2.27(0.41-4.16) | 4.02(1.34-6.76) | 8.50(-13.13-35.5) | 0.04(-0.11-0.20) | 0.17(-0.44-0.79) |
| lag7 | 1.10(0.05-2.16) | 2.37(0.51-4.26) | 3.08(0.46-5.78) | -3.76(-22.6-19.67) | 0.13(-0.02-0.29) | 0.18(-0.43-0.79) |

**Table S11** The lag effect of each 10 μg/m^3^ (0.1 mg/m^3^ for O_3_) increase in pollutants concentration on the number of predicate chronic lower respiratory diseases outpatient volume

| Lagging days | PM_10_  ER (95%CI) | PM_2.5_  ER (95%CI) | NO2  ER (95%CI) | SO2  ER (95%CI) | CO  ER (95%CI) | O3  ER (95%CI) |
| --- | --- | --- | --- | --- | --- | --- |
| lag0 | 1.55(0.37-2.74) | 1.66(-0.17-3.53) | 6.75(3.91-9.66) | 2.41(-21.82-34.13) | 0.02(-0.14-0.18) | -0.18(-0.9-0.54) |
| lag1 | 2.03(0.97-3.10) | 2.05(0.29-3.84) | 6.77(3.91-9.71) | 16.56(-7.16-46.34) | -0.01(-0.16-0.14) | 0.68(0.03-1.34) |
| lag2 | 1.83(0.81-2.87) | 2.05(0.29-3.84) | 5.48(2.86-8.18) | 1.71(-18.3-26.64) | -0.09(-0.24-0.05) | 0.05(-0.57-0.68) |
| lag3 | 2.03(1.02-3.06) | 2.31(0.56-4.08) | 4.16(1.61-6.78) | 34.79(8.69-67.15) | -0.14(-0.28-0.01) | 0.85(0.23-1.48) |
| lag4 | 2.36(1.34-3.40) | 3.31(1.54-5.10) | 6.76(4.13-9.45) | 44.99(17.1-79.51) | -0.03(-0.18-0.12) | 0.73(0.1-1.35) |
| lag5 | 2.21(1.20-3.23) | 2.78(1.02-4.56) | 6.66(4.04-9.34) | 45.57(17.59-80.2) | 0.00(-0.15-0.14) | 0.43(-0.19-1.05) |
| lag6 | 2.39(1.38-3.41) | 3.46(1.71-5.25) | 4.58(1.99-7.24) | 22.78(-1.08-52.41) | 0.00(-0.15-0.14) | 0.67(0.05-1.30) |
| lag7 | 2.01(1.00-3.03) | 3.45(1.70-5.24) | 5.63(3.04-8.29) | 23.42(-0.20-52.63) | 0.12(-0.03-0.26) | 0.76(0.14-1.38) |

**Table S12** The lag effect of each 10 μg/m^3^ (0.1 mg/m^3^ for O_3_) increase in pollutants concentration on the number of internal medicine acute upper respiratory infection outpatient volume

| Lagging days | PM_10_  ER (95%CI) | PM_2.5_  ER (95%CI) | NO2  ER (95%CI) | SO2  ER (95%CI) | CO  ER (95%CI) | O3  ER (95%CI) |
| --- | --- | --- | --- | --- | --- | --- |
| lag0 | 2.86(1.40-4.34) | 2.86(0.66-5.10) | 14.48(10.86-18.21) | 10.13(-21.18-53.89) | 0.02(-0.17-0.21) | -0.06(-0.96-0.85) |
| lag1 | 3.35(2.04-4.68) | 3.13(1.01-5.30) | 11.83(8.23-15.55) | 52.00(14.95-101.00) | -0.04(-0.22-0.14) | 0.48(-0.32-1.29) |
| lag2 | 2.42(1.17-3.70) | 1.73(-0.35-3.86) | 7.11(3.91-10.40) | 33.83(2.25-75.16) | -0.15(-0.32-0.03) | 0.22(-0.55-1.00) |
| lag3 | 1.79(0.54-3.07) | 1.31(-0.78-3.44) | 8.06(4.88-11.34) | 14.19(-12.41-48.86) | -0.12(-0.30-0.06) | -0.38(-1.15-0.39) |
| lag4 | 1.37(0.12-2.65) | 0.55(-1.56-2.70) | 7.62(4.41-10.93) | 4.90(-19.68-37.00) | -0.06(-0.24-0.12) | -0.71(-1.46-0.06) |
| lag5 | 1.12(-0.13-2.39) | 0.50(-1.61-2.66) | 7.29(4.09-10.58) | -4.64(-27.19-24.88) | -0.02(-0.20-0.15) | -0.76(-1.52-0.00) |
| lag6 | 1.27(0.01-2.54) | 1.40(-0.71-3.55) | 8.59(5.40-11.88) | -1.27(-24.48-29.06) | 0.03(-0.15-0.21) | -0.82(-1.58--0.06) |
| lag7 | 0.66(-0.60-1.93) | 1.69(-0.42-3.84) | 7.08(3.90-10.35) | -13.83(-33.93-12.38) | 0.14(-0.04-0.32) | -0.73(-1.49-0.03) |

**Table S13** The lag effect of each 10 μg/m^3^ (0.1 mg/m^3^ for O_3_) increase in pollutants concentration on the number of internal medicine other acute lower respiratory infections outpatient volume

| Lagging days | PM_10_  ER (95%CI) | PM_2.5_  ER (95%CI) | NO2  ER (95%CI) | SO2  ER (95%CI) | CO  ER (95%CI) | O3  ER (95%CI) |
| --- | --- | --- | --- | --- | --- | --- |
| lag0 | 2.86(1.41-4.33) | 5.21(2.89-7.59) | 11.02(7.46-14.69) | 26.95(-9.56-78.18) | 0.10(-0.09-0.29) | 0.86(-0.06-1.78) |
| lag1 | 1.76(0.45-3.08) | 3.00(0.77-5.27) | 9.61(6.04-13.29) | 10.86(-16.86-47.83) | -0.03(-0.21-0.16) | 0.59(-0.23-1.41) |
| lag2 | 1.98(0.73-3.25) | 2.68(0.50-4.92) | 6.56(3.36-9.85) | 31.87(0.58-72.91) | -0.12(-0.3-0.06) | 0.79(0.00-1.59) |
| lag3 | 1.29(0.04-2.57) | 0.6(-1.57-2.81) | 5.22(2.08-8.47) | 2.76(-21.52-34.57) | -0.19(-0.37--0.01) | 0.08(-0.71-0.88) |
| lag4 | 1.4(0.15-2.68) | 1.20(-1.01-3.45) | 4.70(1.53-7.98) | 23.84(-5.43-62.15) | -0.05(-0.23-0.13) | -0.29(-1.06-0.5) |
| lag5 | 1.91(0.66-3.17) | 1.95(-0.25-4.20) | 6.50(3.30-9.80) | 16.80(-11.05-53.36) | -0.06(-0.24-0.12) | -0.25(-1.03-0.54) |
| lag6 | 1.78(0.52-3.04) | 2.72(0.53-4.96) | 6.89(3.71-10.17) | 14.70(-12.43-50.23) | 0.02(-0.16-0.20) | -0.24(-1.02-0.54) |
| lag7 | 1.29(0.04-2.55) | 2.48(0.29-4.72) | 5.06(1.91-8.31) | -7.52(-29.28-20.93) | 0.02(-0.15-0.20) | -0.02(-0.79-0.76) |

**Table S14** The lag effect of each 10 μg/m^3^ (0.1 mg/m^3^ for O_3_) increase in pollutants concentration on the number of internal medicine influenza and pneumonia outpatient volume

| Lagging days | PM_10_  ER (95%CI) | PM_2.5_  ER (95%CI) | NO2  ER (95%CI) | SO2  ER (95%CI) | CO  ER (95%CI) | O3  ER (95%CI) |
| --- | --- | --- | --- | --- | --- | --- |
| lag0 | 1.61(-0.64-3.91) | 1.07(-2.31-4.57) | 8.60(3.13-14.35) | 29.04(-24.97-121.94) | 0.06(-0.24-0.36) | -0.25(-1.63-1.14) |
| lag1 | -0.71(-2.71-1.32) | -2.35(-5.52-0.93) | 6.63(1.18-12.37) | 12.25(-29.52-78.75) | -0.15(-0.43-0.13) | 0.05(-1.20-1.33) |
| lag2 | -1.29(-3.23-0.68) | -2.76(-5.9-0.48) | 3.72(-1.21-8.89) | -8.34(-41.26-43.02) | -0.07(-0.35-0.2) | -0.71(-1.92-0.53) |
| lag3 | -0.55(-2.5-1.44) | -2.17(-5.32-1.08) | 2.53(-2.25-7.55) | -1.59(-36.57-52.68) | 0.01(-0.26-0.29) | 0.10(-1.13-1.34) |
| lag4 | 1.05(-0.9-3.05) | 1.75(-1.51-5.12) | 4.13(-0.81-9.32) | 48.58(-4.27-130.62) | 0.03(-0.24-0.31) | 0.16(-1.04-1.38) |
| lag5 | 0.32(-1.58-2.26) | -1.34(-4.53-1.95) | 2.51(-2.3-7.57) | 34.97(-13.19-109.84) | -0.15(-0.43-0.14) | 0.01(-1.18-1.22) |
| lag6 | 1.14(-0.77-3.10) | 1.00(-2.19-4.29) | 3.10(-1.73-8.17) | 57.36(2.07-142.62) | -0.11(-0.39-0.17) | -0.14(-1.34-1.07) |
| lag7 | 0.93(-1.00-2.90) | 0.73(-2.46-4.02) | 2.75(-2.09-7.82) | 11.53(-27.76-72.2) | 0.06(-0.22-0.33) | -0.25(-1.46-0.96) |

**Table S15** The lag effect of each 10 μg/m^3^ (0.1 mg/m^3^ for O_3_) increase in pollutants concentration on the number of internal medicine other upper respiratory tract diseases outpatient volume

| Lagging days | PM_10_  ER (95%CI) | PM_2.5_  ER (95%CI) | NO2  ER (95%CI) | SO2  ER (95%CI) | CO  ER (95%CI) | O3  ER (95%CI) |
| --- | --- | --- | --- | --- | --- | --- |
| lag0 | 2.38(1.29-3.47) | 2.35(0.74-3.99) | 10.39(7.79-13.06) | 3.04(-19.2-31.41) | 0.00(-0.14-0.14) | 0.15(-0.51-0.81) |
| lag1 | 2.59((1.62-3.57) | 2.88(1.31-4.46) | 9.94(7.32-12.62) | 19.89(-2.37-47.24) | -0.02(-0.15-0.11) | 0.5(-0.08-1.1) |
| lag2 | 2.64(1.71-3.59) | 3.42(1.87-5.00) | 8.86(6.46-11.31) | 28.57(5.66-56.46) | -0.07(-0.2-0.05) | 0.51(-0.06-1.08) |
| lag3 | 2.81(1.89-3.75) | 3.46(1.91-5.03) | 9.22(6.85-11.64) | 33.89(10.45-62.32) | -0.06(-0.2-0.06) | 0.65(0.07-1.22) |
| lag4 | 2.88(1.95-3.82) | 3.62(2.06-5.20) | 9.76(7.37-12.20) | 42.10(17.31-72.12) | -0.04(-0.18-0.08) | 0.51(-0.04-1.08) |
| lag5 | 2.49(1.56-3.44) | 2.93(1.36-4.52) | 8.66(6.28-11.10) | 31.72(8.6-59.78) | -0.03(-0.17-0.09) | 0.33(-0.23-0.9) |
| lag6 | 2.45(1.52-3.39) | 3.53(1.97-5.11) | 7.97(5.60-10.40) | 26.28(4.05-53.26) | 0.00(-0.13-0.12) | 0.37(-0.19-0.94) |
| lag7 | 2.19(1.25-3.13) | 3.50(1.94-5.08) | 7.92(5.55-10.35) | 21.06(0.02-46.52) | 0.03(-0.09-0.16) | 0.35(-0.21-0.92) |

**Table S16** The lag effect of each 10 μg/m^3^ (0.1 mg/m^3^ for O_3_) increase in pollutants concentration on the number of internal medicine chronic lower respiratory diseases outpatient volume

| Lagging days | PM_10_  ER (95%CI) | PM_2.5_  ER (95%CI) | NO2  ER (95%CI) | SO2  ER (95%CI) | CO  ER (95%CI) | O3  ER (95%CI) |
| --- | --- | --- | --- | --- | --- | --- |
| lag0 | 1.22(-0.01-2.46) | 0.97(-0.89-2.86) | 8.98(6.01-12.04) | 2.00(-23.18-35.42) | 0.02(-0.14-0.18) | -0.09(-0.81-0.63) |
| lag1 | 0.66(-0.44-1.77) | -0.31(-2.09-1.5) | 8.39(5.38-11.48) | 3.92(-18.04-31.77) | -0.04(-0.19-0.11) | -0.36(-1-0.29) |
| lag2 | 0.80(-0.25-1.86) | 0.24(-1.51-2.03) | 3.87(1.20-6.61) | 6.71(-14.86-33.73) | -0.12(-0.27-0.03) | -0.01(-0.64-0.62) |
| lag3 | 1.37(0.33-2.43) | 0.9(-0.86-2.69) | 4.26(1.63-6.96) | 27.33(2.17-58.67) | -0.11(-0.26-0.04) | 0.12(-0.51-0.75) |
| lag4 | 1.39(0.34-2.45) | 1.19(-0.61-3.02) | 4.64(1.97-7.39) | 12.28(-10.08-40.21) | -0.05(-0.2-0.11) | -0.22(-0.84-0.41) |
| lag5 | 1.02(-0.02-2.06) | 0.46(-1.32-2.27) | 3.05(0.41-5.75) | 4.99(-16.15-31.44) | -0.1(-0.25-0.06) | -0.59(-1.2-0.03) |
| lag6 | 1.11(0.07-2.16) | 1.82(0.05-3.62) | 3.18(0.56-5.87) | 7.07(-14.41-33.95) | -0.03(-0.18-0.12) | -0.53(-1.14-0.09) |
| lag7 | 0.74(-0.29-1.79) | 0.29(-1.45-2.06) | 3.96(1.32-6.66) | -3.55(-22.63-20.24) | 0.00(-0.15-0.15) | -0.46(-1.07-0.15) |

**Figure S1** The Daily Average Concentration Trends of PM_10_(A)、PM_2.5_(B)、NO_2_(C)、SO_2_(D)、CO(E)andO_3_-8h(F) in Fuzhou City.


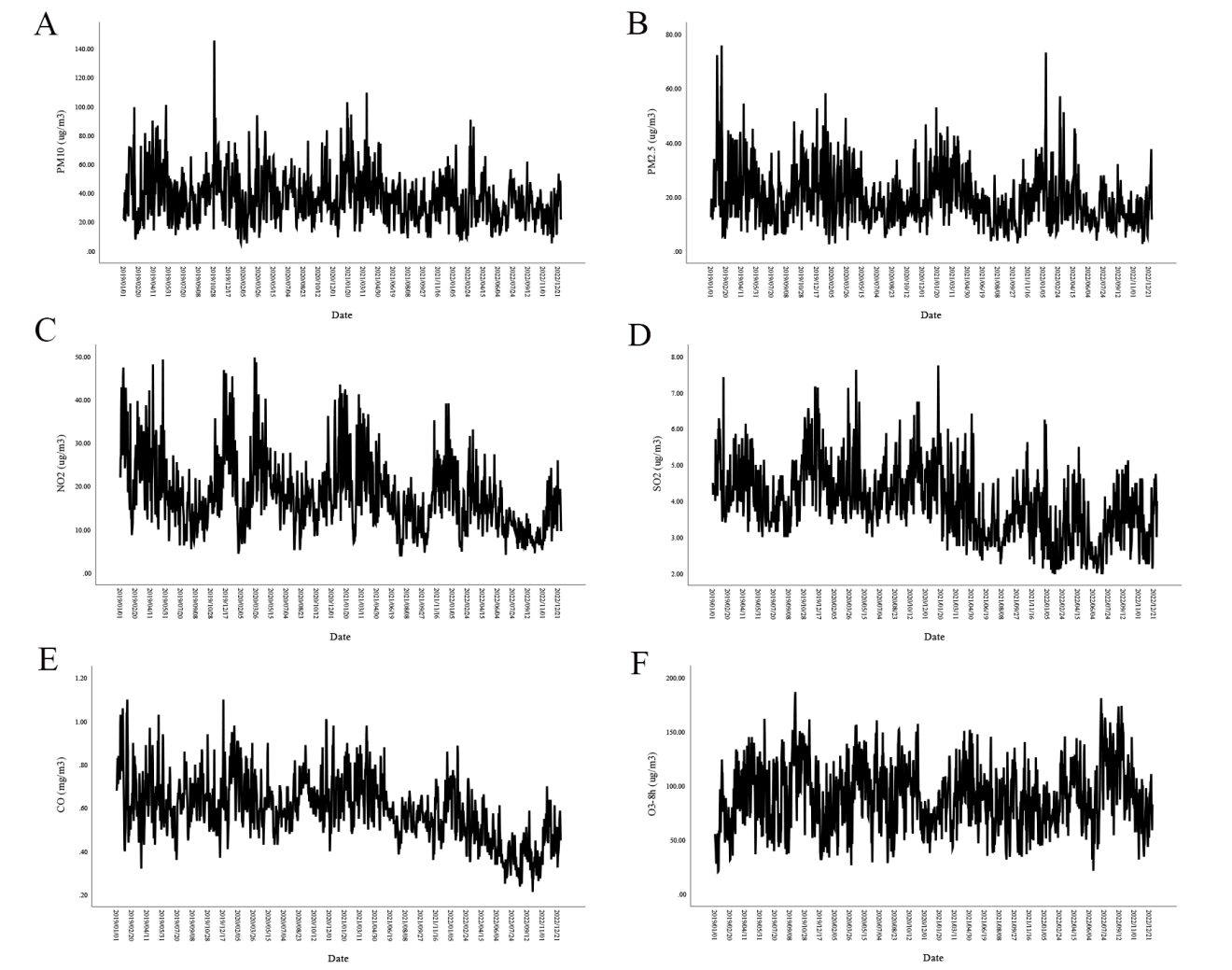


**Figure S2** The Daily Average Trends of Temperature(A), Pressure(B), Relative Humidity(C) and Wind Speed(D) in Fuzhou City.


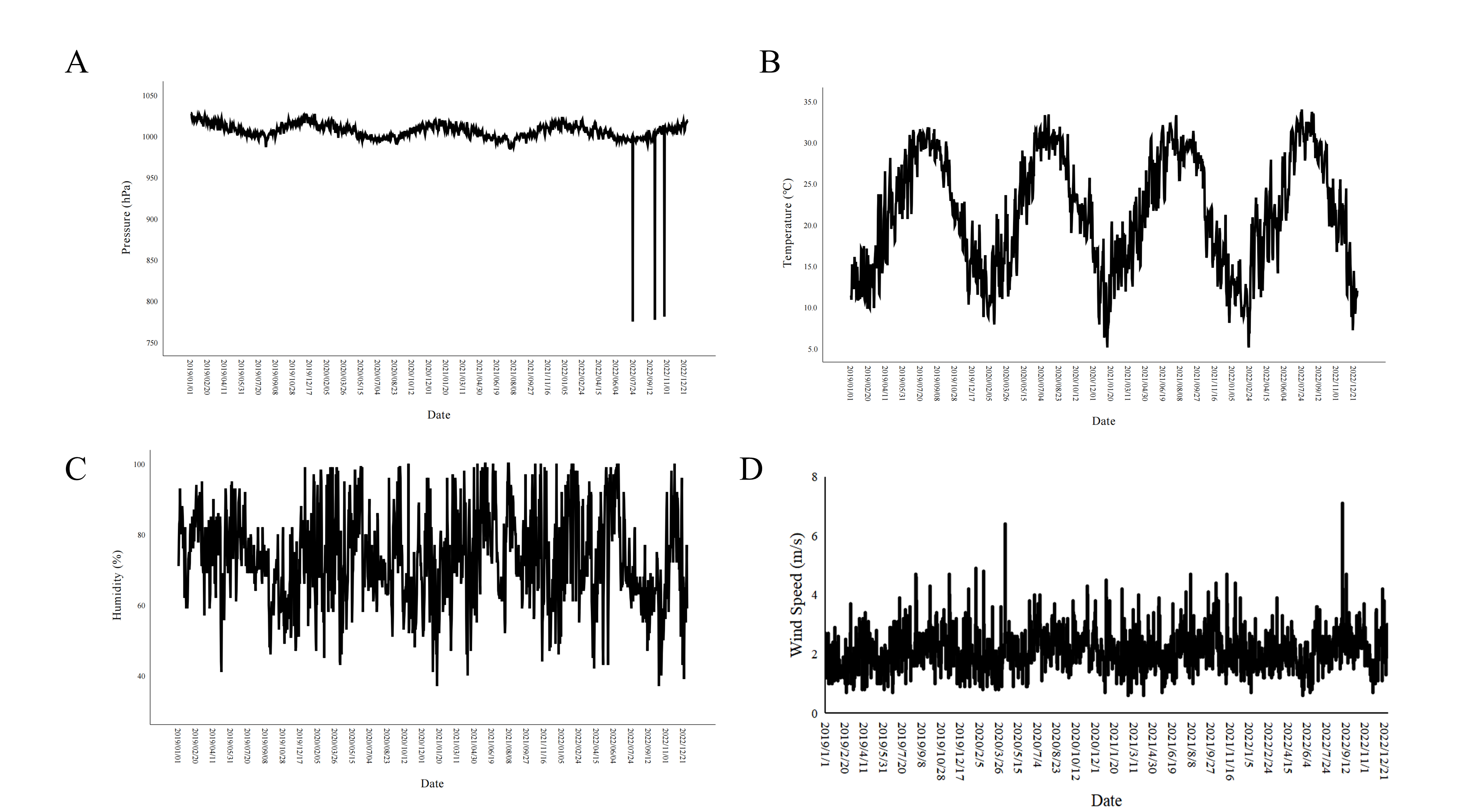


**Figure S3 Lagged effects of different air pollutants on predicate acute upper respiratory infection outpatient volume (A. PM_2.5_, B. PM_10_, C. CO, D. NO_2_, E. SO_2_, F. O_3_).**

**
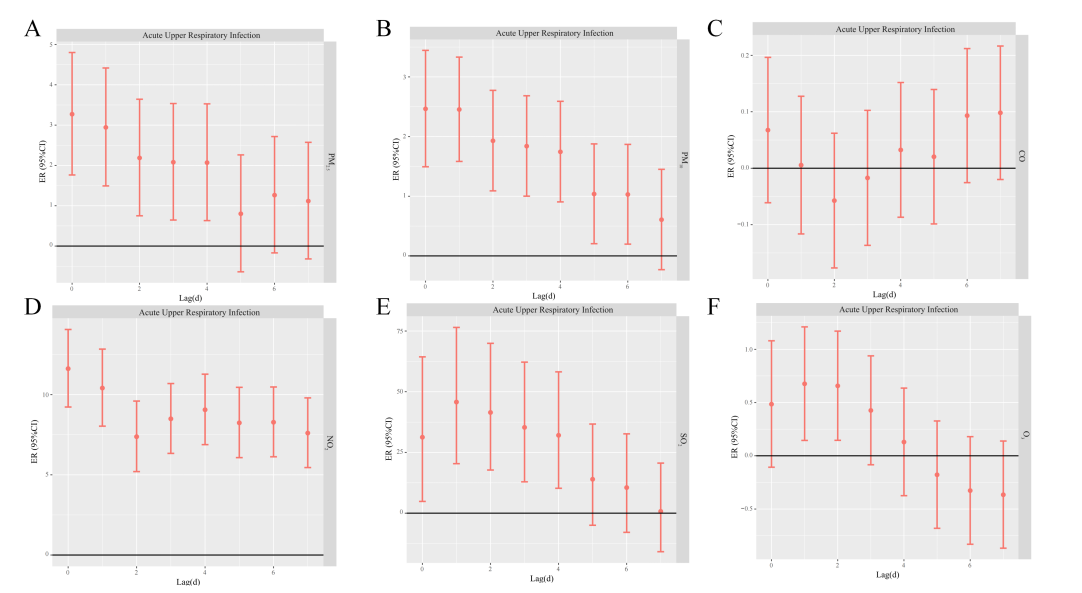
**

**Figure S4 Lagged effects of different air pollutants on pediatric influenza and pneumonia outpatient volume (A. PM_2.5_, B. PM_10_, C. CO, D. NO_2_, E. SO_2_, F. O_3_).**

**
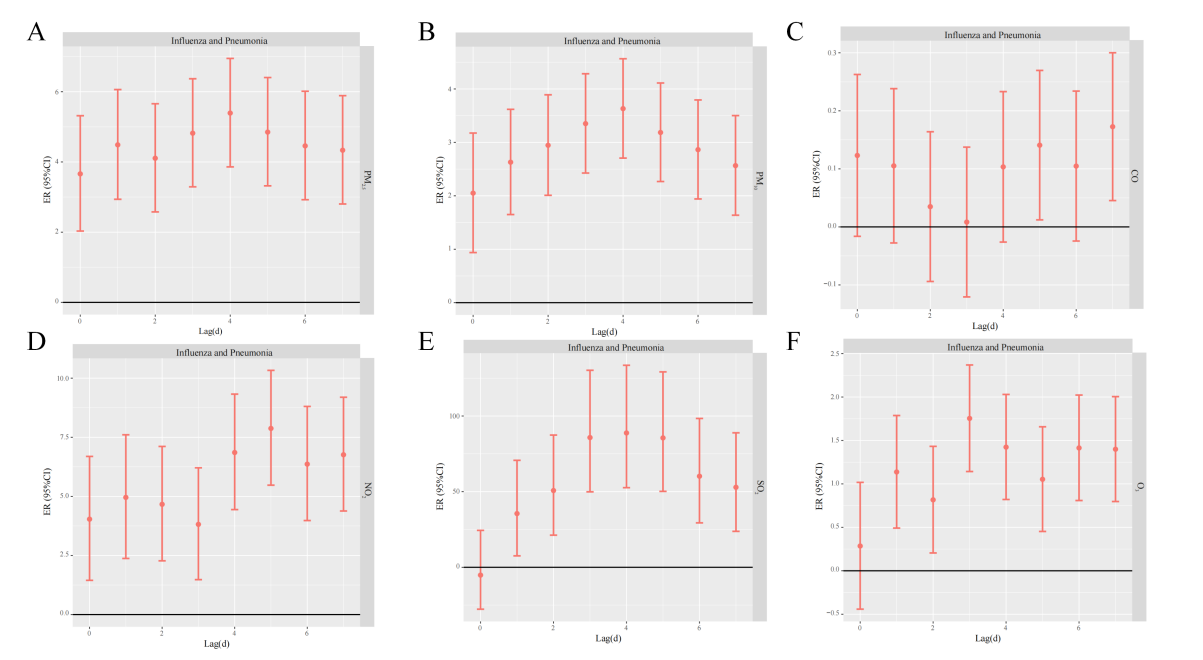
**

**Figure S5 Lagged effects of different air pollutants on pediatric other acute lower respiratory Infections outpatient volume (A. PM_2.5_, B. PM_10_, C. CO, D. NO_2_, E. SO_2_, F. O_3_).**

**
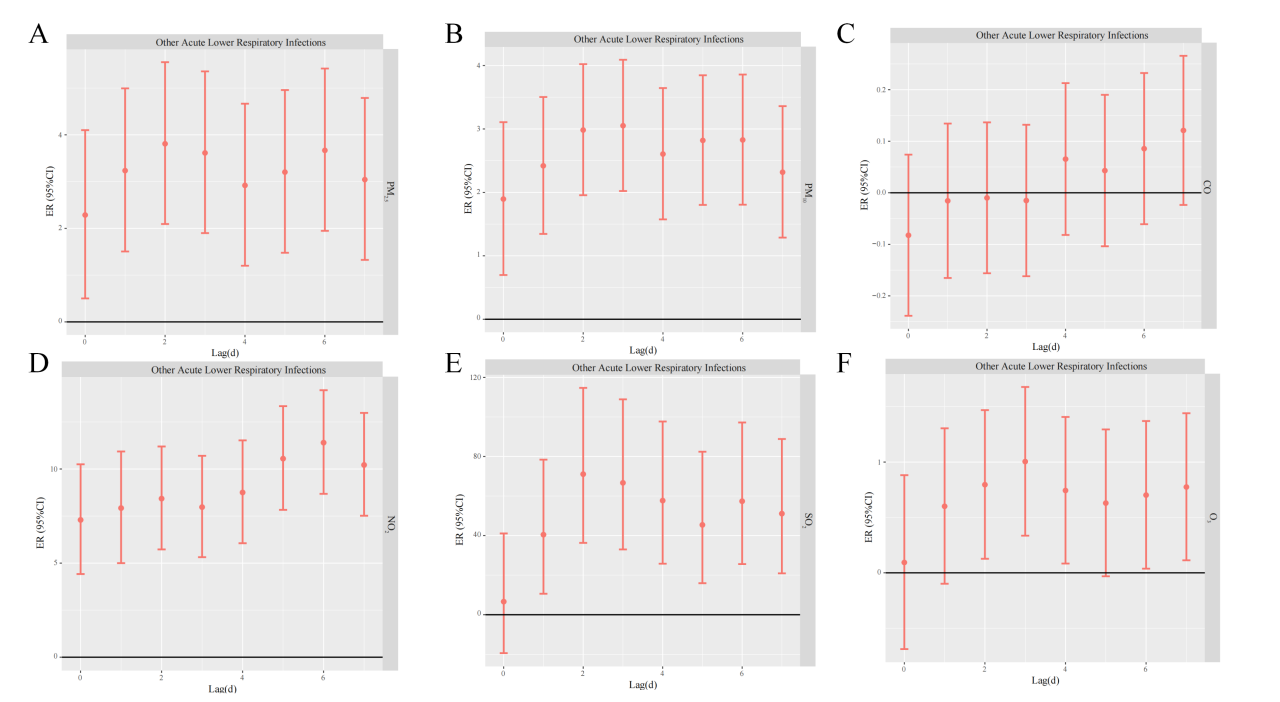
**

**Figure S6 Lagged effects of different air pollutants on pediatric other upper respiratory tract diseases outpatient volume(A. PM_2.5_, B. PM_10_, C. CO, D. NO_2_, E. SO_2_, F. O_3_).**

**
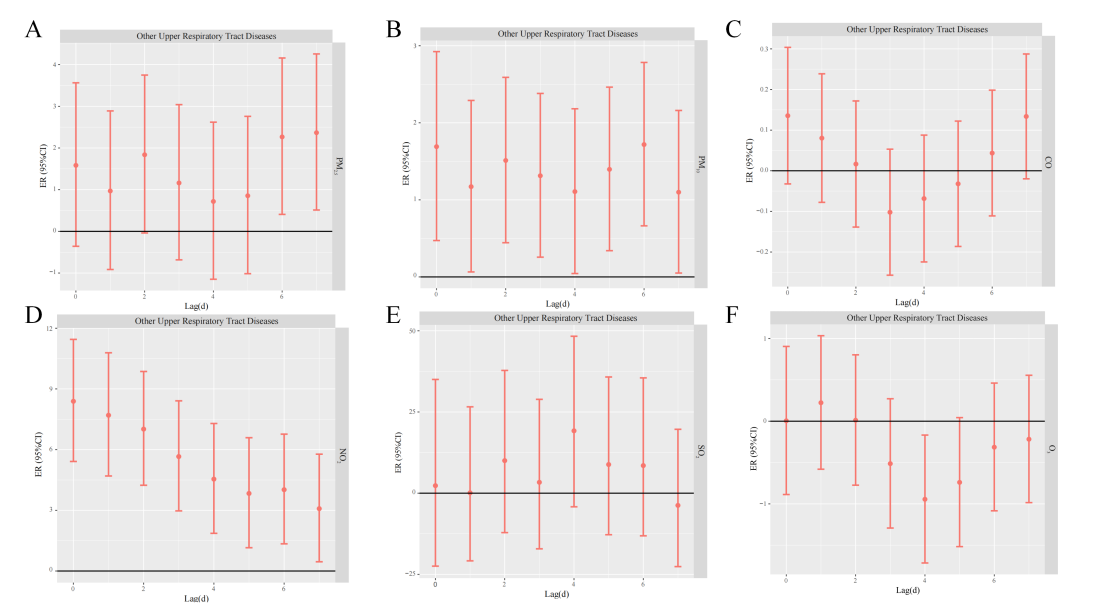
**

**Figure S7 Lagged effects of different air pollutants on predicate chronic lower respiratory diseases outpatient volume (A. PM_2.5_, B. PM_10_, C. CO, D. NO_2_, E. SO_2_, F. O_3_).**


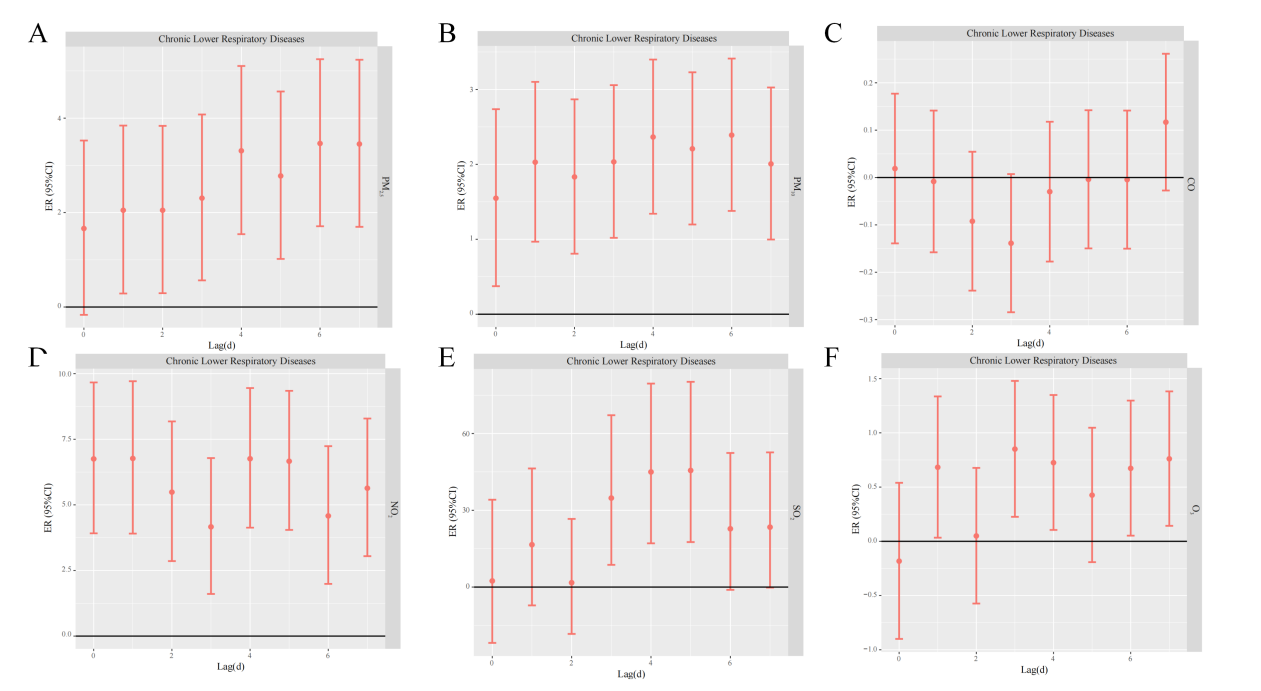


**Figure S8 Lagged effects of different air pollutants on internal medicine acute upper respiratory infection outpatient volume (A. PM_2.5_, B. PM_10_, C. CO, D. NO_2_, E. SO_2_, F. O_3_).**

**
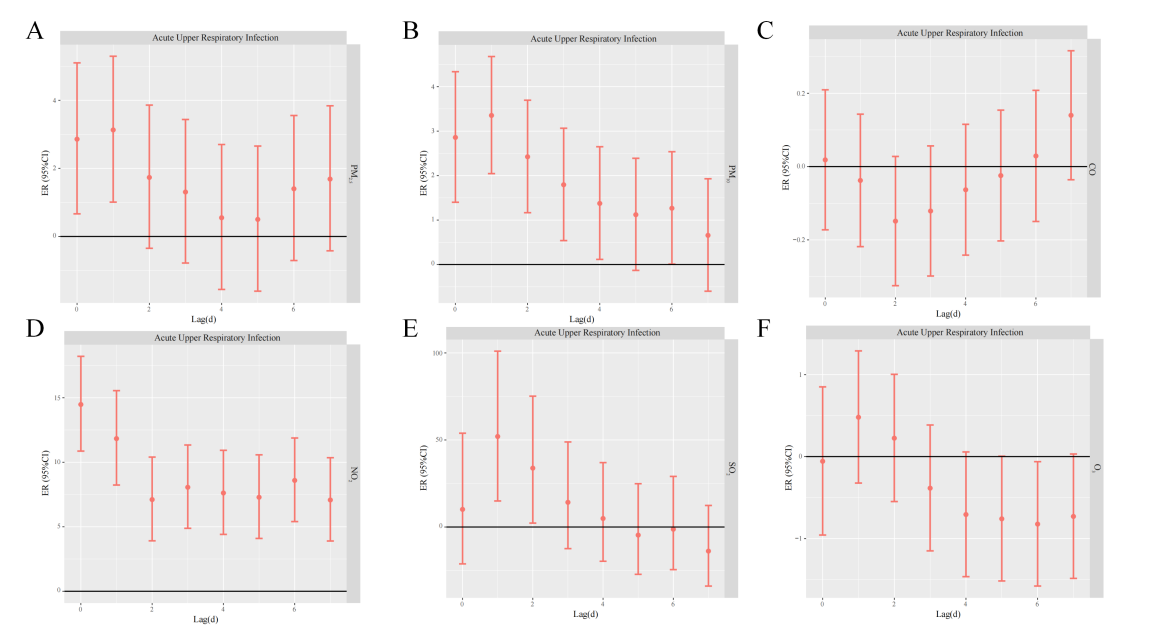
**

**Figure S9 Lagged effects of different air pollutants on internal medicine other acute lower respiratory infections outpatient volume (A. PM_2.5_, B. PM_10_, C. CO, D. NO_2_, E. SO_2_, F. O_3_).**

**
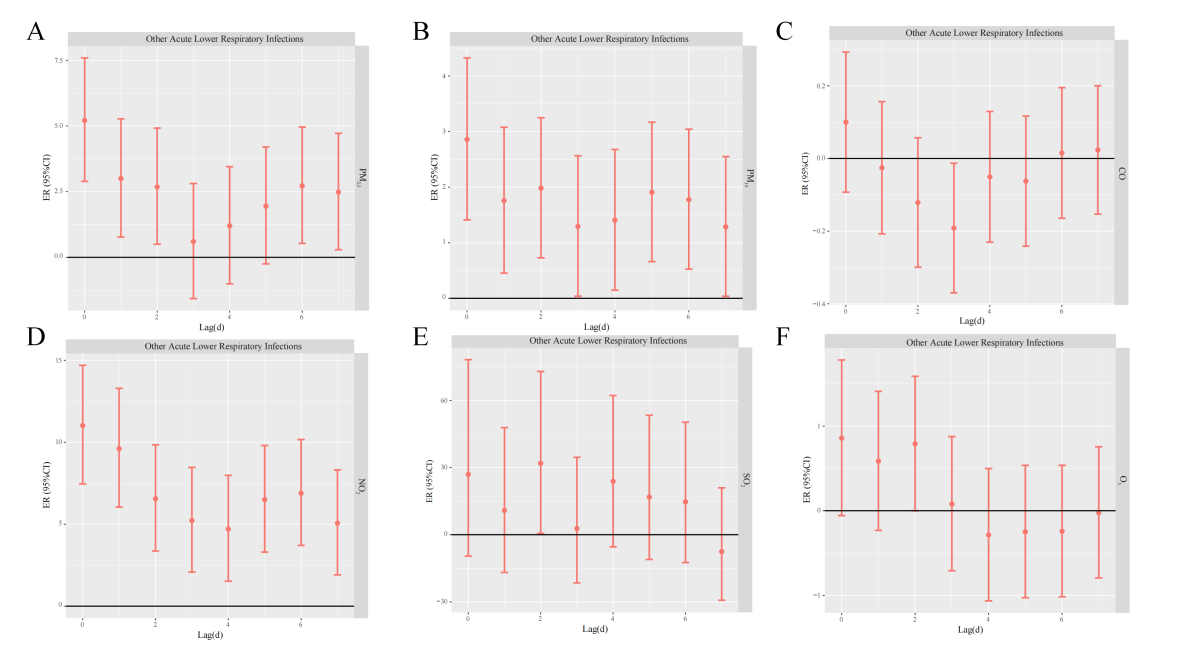
**

**Figure S10 Lagged effects of different air pollutants on internal medicine influenza and pneumonia outpatient volume (A. PM_2.5_, B. PM_10_, C. CO, D. NO_2_, E. SO_2_, F. O_3_).**

**
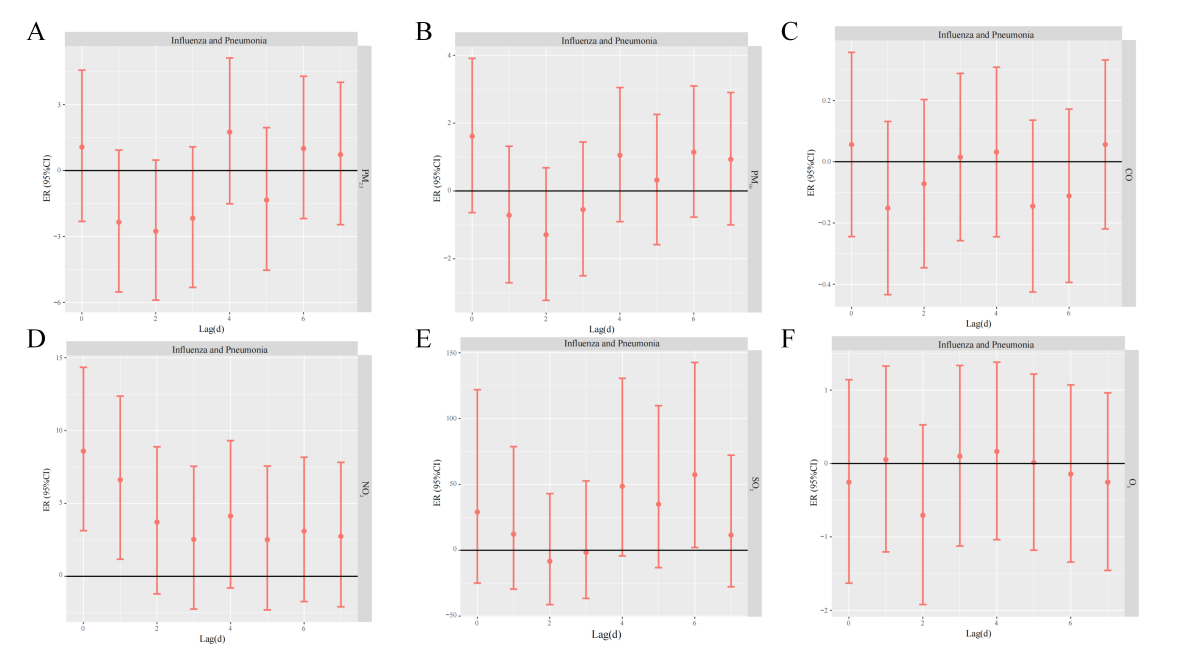
**

**Figure S11 Lagged effects of different air pollutants on internal medicine other upper respiratory tract diseases outpatient volume (A. PM_2.5_, B. PM_10_, C. CO, D. NO_2_, E. SO_2_, F. O_3_).**

**
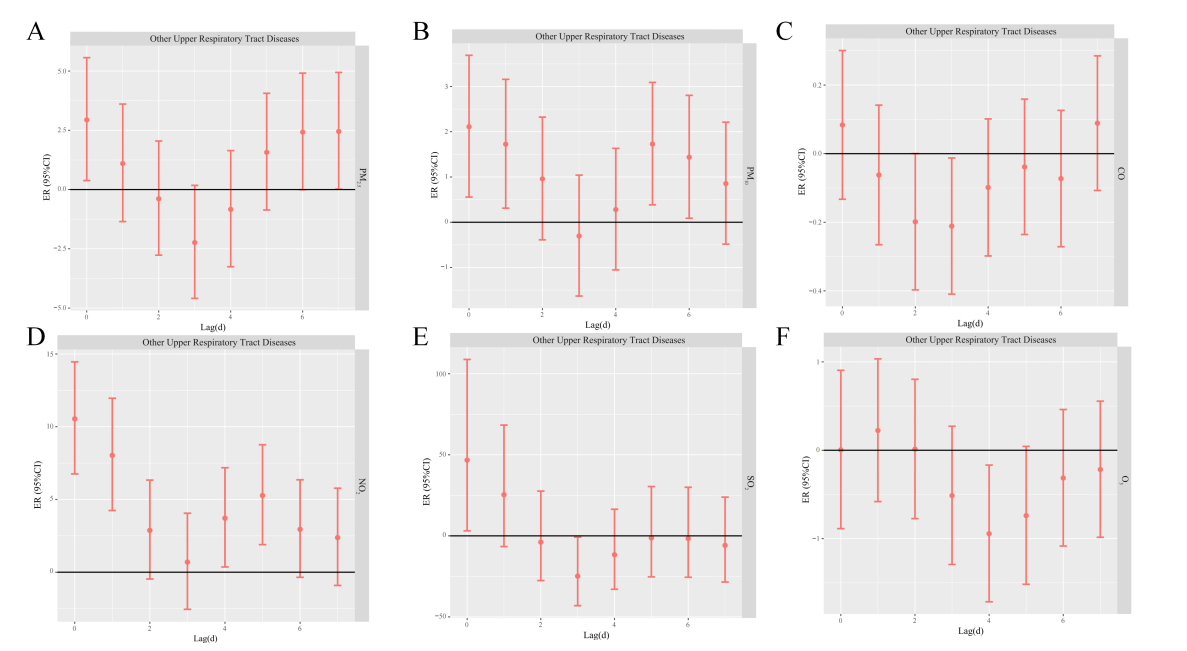
**

**Figure S12 Lagged effects of different air pollutants on internal medicine chronic lower respiratory diseases outpatient volume (A. PM_2.5_, B. PM_10_, C. CO, D. NO_2_, E. SO_2_, F. O_3_).**


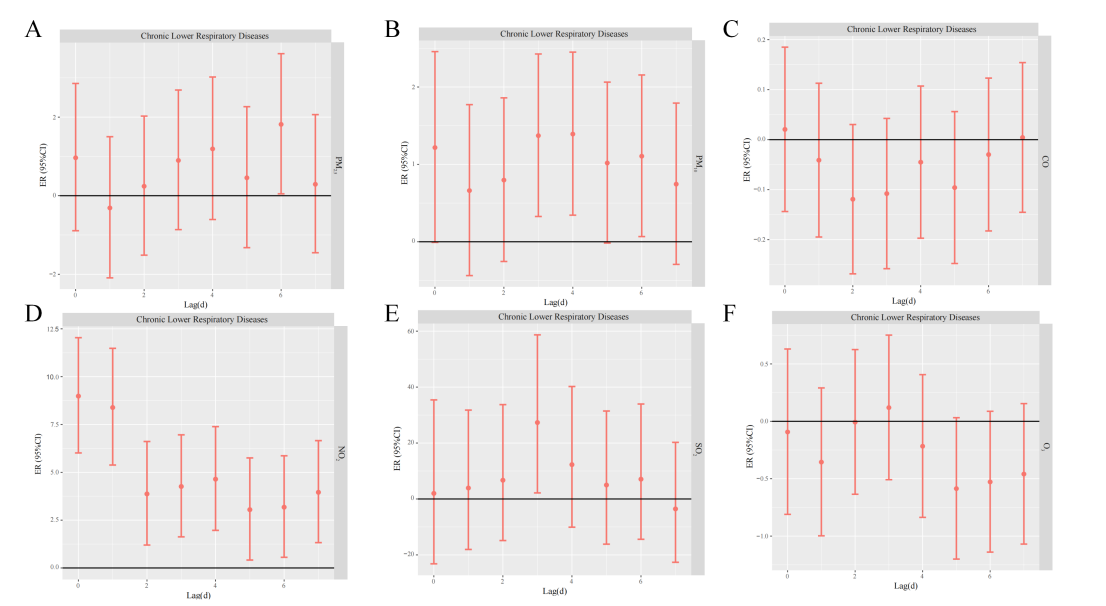


**Figure S13 Sensitivity analysis of the lagged effect of different air pollutants on respiratory outpatient volume (A. PM_2.5_, B. PM_10_, C. CO, D. NO_2_, E. SO_2_, F. O_3_).**

**
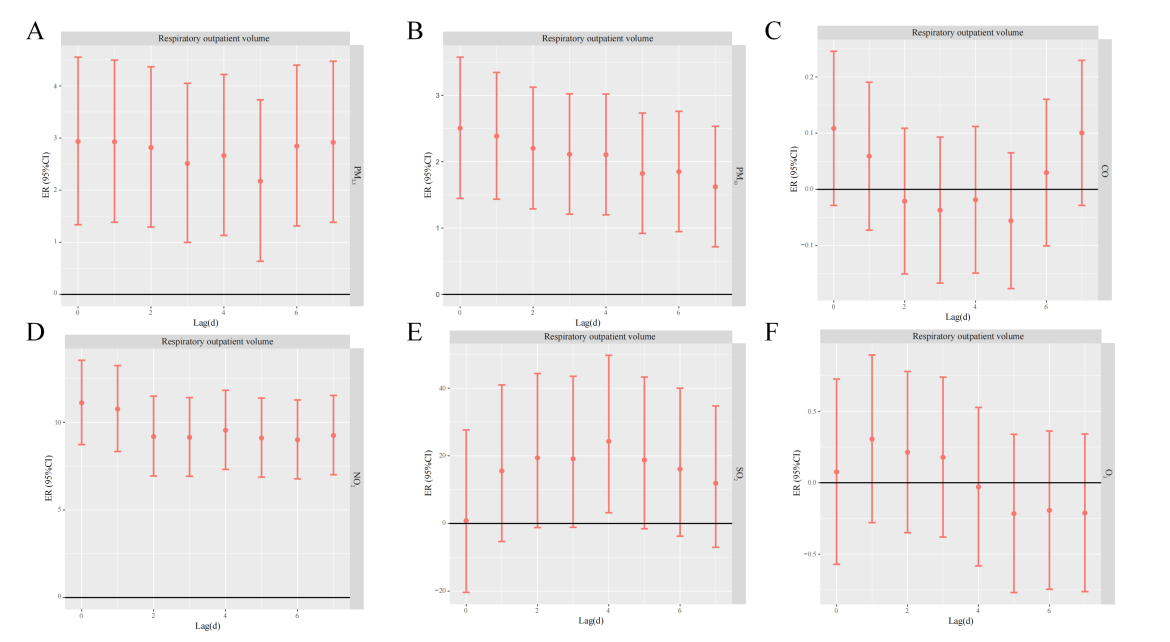
**

**Figure S14 Sensitivity analysis of the lagged effect of different air pollutants on pediatric respiratory outpatient volume (A. PM_2.5_, B. PM_10_, C. CO, D. NO_2_, E. SO_2_, F. O_3_).**

**
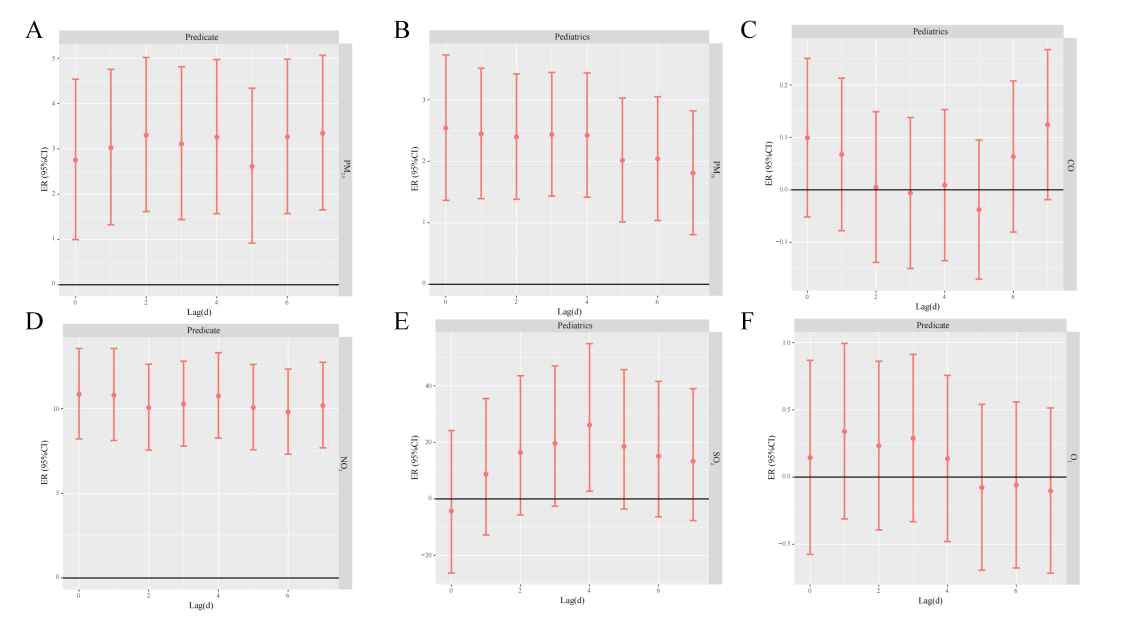
**

**Figure S15 Sensitivity analysis of the lagged effect of different air pollutants on the volume of internal medicine respiratory outpatient volume (A. PM_2.5_, B. PM_10_, C. CO, D. NO_2_, E. SO_2_, F. O_3_).**

**
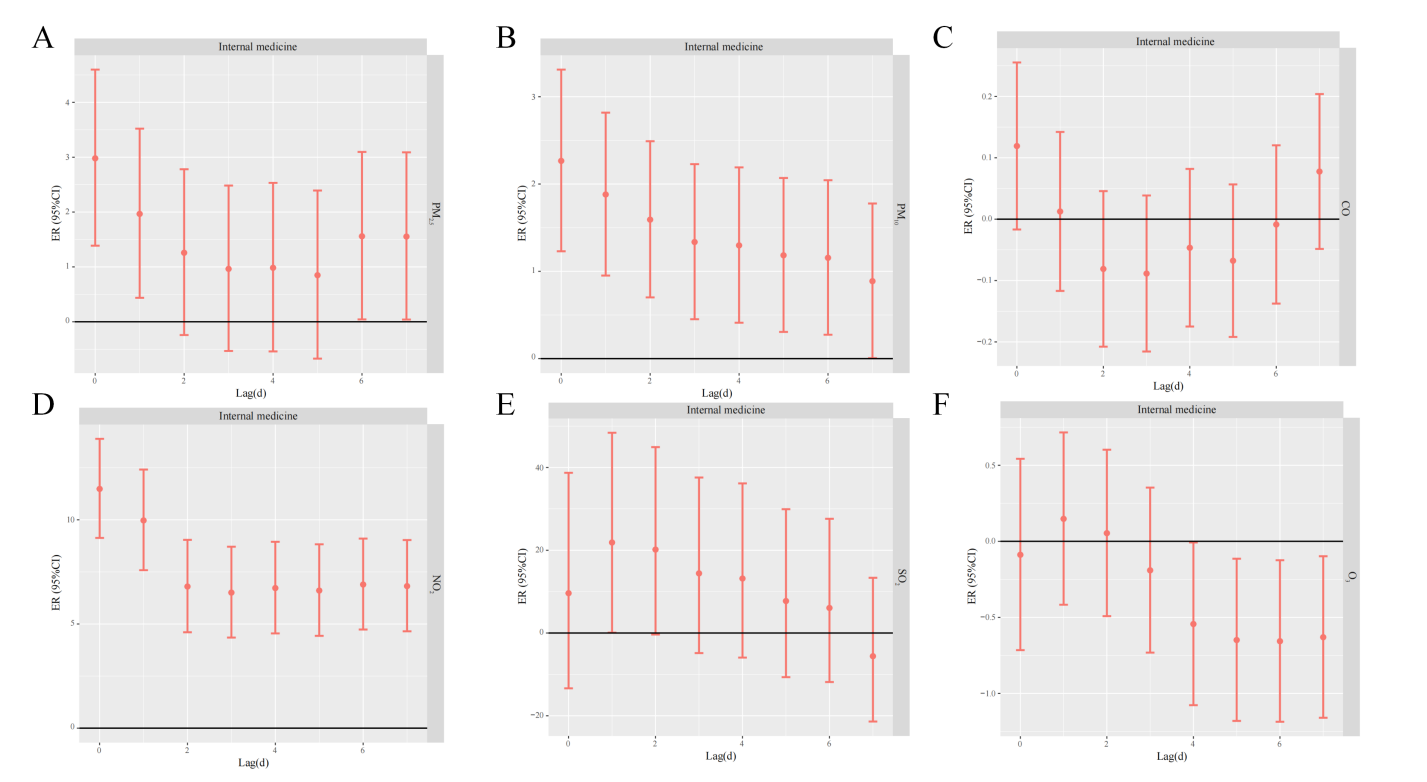
**
